# Identifying treponemal disease in early East Asia

**DOI:** 10.1002/ajpa.24526

**Published:** 2022-04-27

**Authors:** Yawei Zhou, Guoshuai Gao, Xiangyu Zhang, Bo Gao, Chenggang Duan, Hong Zhu, Aida R. Barbera, Siân Halcrow, Kate Pechenkina

**Affiliations:** ^1^ College of History Zhengzhou University Zhengzhou Henan China; ^2^ School of Archaeology Jilin University Changchun Jilin China; ^3^ Xi'an Institute of Cultural Relics Protection and Archaeology Xian Shanxi China; ^4^ Université Laval Québec Quebec Canada; ^5^ Department of Anthropology Queens College of the City University of New York Queens New York USA; ^6^ University of Otago Dunedin New Zealand

**Keywords:** paleopathology, skeletal lesions, tang dynasty, *Treponema pallidum*

## Abstract

**Objectives:**

Historic records suggest that a virulent form of treponematosis, sexually transmitted syphilis was introduced to Asia from Europe by the da Gama crew, who landed in India in 1498. Our objective is to assess the gross pathology of human skeletal remains from the Tang dynasty of China to test the presence of treponemal infection in East Asia before 1498. We interpret this paleopathological evidence in the context of site ecology and sociocultural changes during the Tang dynasty.

**Materials and methods:**

We examined the gross pathology of 1598 human skeletons from Xingfulindai (AD 618 to AD 1279) archeological site located on the Central Plain of China. Using the modified diagnostic criteria defined by Hackett's classical work, we classify the pathology as consistent, strongly suggestive, or pathognomonic for treponemal infection.

**Results:**

Twelve adult individuals from Xingfulindai had bone lesions suggestive of systemic pathology. Two of these individuals displayed a combination of lesion patterns pathognomonic of treponemal disease and one had lesions consistent with treponematosis. The radiocarbon dates for the bone samples from these skeletons place them before AD 1200.

**Conclusions:**

The location of Xingfulindai in a continental climatic zone is not typical for yaws and bejel ecology, because these strains occur in the tropics, or in hot, dry environments, respectively. The urban setting, where there is documented evidence for increased interaction between multiple ethnic groups and a developed institution of courtesans during the Tang dynasty, favors sexually transmitted syphilis as the more likely diagnosis. This study supports an earlier spread of syphilis to China than 1498.

## Graphical abstract

Syphilis in China before Vasco da Gama?

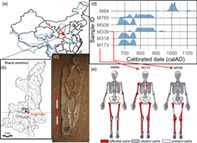

## 1 INTRODUCTION

Sexually transmitted syphilis (ST syphilis), yaws, and endemic syphilis (bejel) are caused by three subspecies of the spirochete bacteria *Treponema pallidum*: *T. p. ssp pallidum*, *T. p. ssp pertenue*, and *T. p. ssp endemicum*, respectively. Together with the tropical skin disease pinta, linked to *T. carateum*, these infectious diseases are collectively referred to as treponematoses (Baker et al., 2020; Buckley & Oxenham, 2016; Fu, 2009; Gogarten et al., 2016; Hudson, 1965). Because there is remarkable genetic similarity among the three strains of *Treponema pallidum* (Gogarten et al., 2016), their manifestations, mortality rates, routes of transmission, and infectivity overlap considerably. The sexual route of transmission is the most typical for ST syphilis, while the others are passed on by skin‐to‐skin contact or from insects or via both routes. Occasional sexual transmission of yaws and contact transmission of ST syphilis has been documented. On a molecular level, there is no clear understanding of whether human‐specific treponema co‐diverged with their primate hosts through the course of human evolution and hence have always been harbored by our species, have crossed species boundaries once or multiple times, or persist in zoonotic reservoirs (Gogarten et al., 2016). Baker et al. (2020:11‐12) notes multiple gaps in available *T. pallidum* genomes preclude clear understanding of the geographic origin and spread of treponematosis.

**FIGURE 1:**
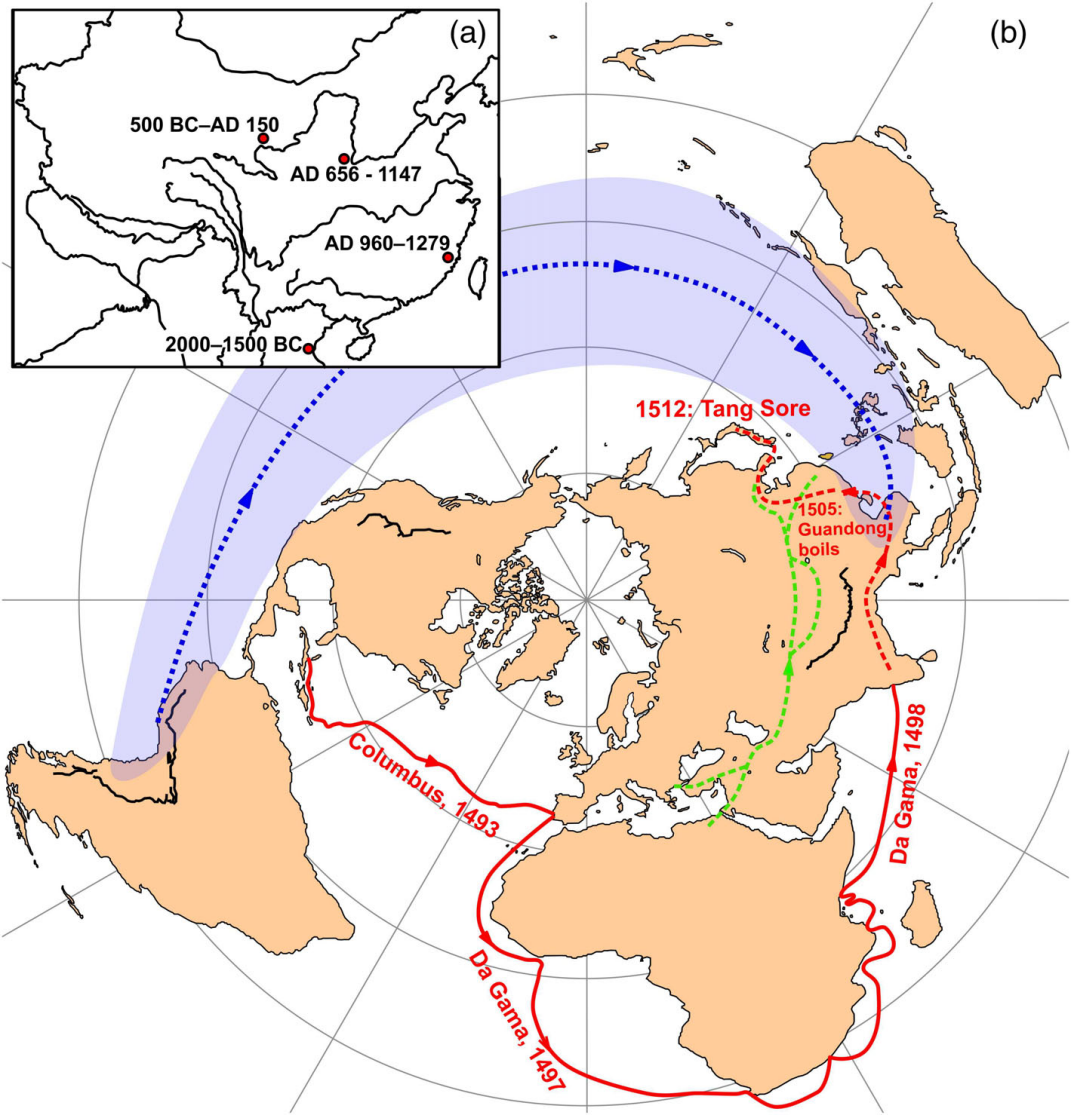
Vasco da Gama and pre‐da Gama models of treponemal spread to East Asia. (a) Possible early skeletal cases of the treponemal disease in China, including Qinghai Province, near Xining City (Kayue culture) (500 BC–AD 150) (Suzuki et al., 2005); on a skeleton from Fujian Province dated to the Song dynasty (AD 960–1279) (Winterbottom, 2014); and the Xingfulindai tomb area, located in the suburb of Xi'an City, Shaanxi Province (AD 656–1147) (present study). Two probable skeletal cases from the Man Bac site in Ninh Binh Province, Vietnam (ca 2000–1500 BC) (Vlok et al., 2020). (b) The three hypothesis of treponematosis arrival to China: Red line—the out of America spread by Columbus's crewmen to Europe followed by the Vaso da Gama's voyage to India in 1498, to China via Southeast Asia by 1505, and to Japan by 1512, based on historic records; green line: a possible earlier spread via trade route network from Africa and Middle East; blue line: a possible earlier spread out of America via the Pacific to Micronesia and from there to Polynesia, Southeast Asia and China

Historically, three alternative models for the introduction of treponemal infection to continental China can be found in the literature: the Vasco da Gama model (Bi, 2007; Fu, 2009; Wang, 1997), the Trade Road model (Suzuki et al., 2005) and the Pacific model (Buckley & Oxenham, 2016) (Figure 1). The Vasco da Gama model is equivalent to the Columbian model from Europe (Armelagos et al., 2012; Baker et al., 2020; Crosby, 1969; Harper et al., 2011; Harrison, 1959; Hudson, 1968; Tampa et al., 2014). It posits that syphilis was introduced to South Asia by Vasco da Gama's expedition in 1498, from where it spread to Southeast Asia and then to the coast of China (Fu, 2009; Wong & Wu, 1936). The first recorded case of the “boils of Guandong,” as syphilis was known, dates to 1505 from Guangzhou, the main port of contact with Europeans. By 1512 the disease had reached Japan, where it was referred to as “Tang sore 唐瘡” and “Ryukyu sore 琉球瘡” (Suzuki, 2001), pointing at cultural perceptions of the disease as alien. The Ryukyu Islands were not fully incorporated into the Japanese empire until 19th century and were perceived as external to Japan through much of its history. The da Gama model stems from the apparent absence of any mention of sexually transmitted diseases in early medical texts from China before the Ming dynasty, despite the wealth of textual evidence for other infectious diseases, including leprosy (Bi, 2007; Gwei‐Djen & Needham, 1993; Harper, 1990; Zhang, 2004). Based on two possible skeletal cases of early syphilis from northeastern China, Suzuki et al. (2005) proposed that this disease was introduced via the trade road network that connected Africa, Middle East and Europe.

An independent spread of syphilis to East Asia through the Pacific has also been proposed (Buckley & Oxenham, 2016). Vlok et al. (2020) have recently published evidence arguing for probable *T. p. ssp pertenue* (yaws) in Vietnam about 4000 years ago. They consider the migration of farmers from Southern China into the region as a route of transmission for this disease. Finally, it is conceivable that different forms of treponemal diseases have been present for a long time but evaded historic documentation in some regions by being confused with other diseases (Figure 1). Previously isolated regions were brought into contact by these trade routes, leading to the emergence of large multiethnic urban centers through these relationships (Waugh, 2010).

Baker et al.’s (2020) recent overview of the mounting paleopathological and genetic evidence for treponemal infections through early human history, called to move the debate of the origin of treponematosis away from the pre‐Columbian vs. Columbia dichotomy and explore under what circumstances different related diseases caused by subspecies of *Treponema pallidum* emerged throughout the world. Findings of treponemal infection in African non‐human primates (Baylet et al., 1971; Fribourg‐Blanc et al., 1966; Harper et al., 2012; Knauf et al., 2012; Knauf et al., 2013; Levréro et al., 2007; Lovell et al., 2000) support the possibility of an African origin of human treponematosis. It is conceivable that multiple cross‐species transmissions of pathogenic *Treponema* occurred in Africa in the past or even that African primates continue to serve as a zoonotic reservoir for human yaws (Knauf et al., 2013). This interpretation of an African origin is supported by the greater genetic divergence among *Treponema* strains infecting baboons in different parts of Africa as compared to *Treponema* strains causing yaws isolated from contemporary humans (Harper et al., 2012), making the existence of a primate reservoir in the past also plausible. The high genetic similarity of *T. pallidum* to a rabbit pathogen *T. paraluiscuniculi* has also been reported, suggesting a possibility of anthroponotic transmission of human *Treponema* to animals (Šmajs et al., 2011). Similarly, an anthroponotic transmission of *Treponema* to African non‐human primates cannot be ruled out.

The molecular phylogeny of the pathogenic *Treponema* is notoriously difficult to construct (Baker et al., 2020). Because the different strains recombine and due to the difficulties of isolating sufficiently long sequences of treponemal DNA from archeological skeletal samples, phylogenetic analysis remains impossible for the time being. Complete genomes of *Treponema pallidum sub. pertenue* extracted from three 16th‐century African individuals uncovered from a mass grave in Mexico had strains that were similar to those affecting current West African populations, which seems to support an African origin of this strain (Barquera et al., 2020). Recent studies examining genomic diversity of syphilis strains in modern China (Chen et al., 2021) add valuable datapoints to our understanding of syphilis molecular phylogeny and may eventually lead to a better understanding of *Treponema* evolution and the origins and timing of early epidemics, when combined with the studies on modern or early genomes from other parts of the world (Giffin et al., 2020; Majander et al., 2020). Until full genome sequences of *Treponema* strains from earlier archeologically derived human bone samples become available, identification of human skeletons with lesions indicative of treponemal disease remains our primary approach for reconstructing the origin and early epidemiology of this important infection.

## 2 MATERIALS AND METHODS

### 2.1 Archeological background

**FIGURE 2:**
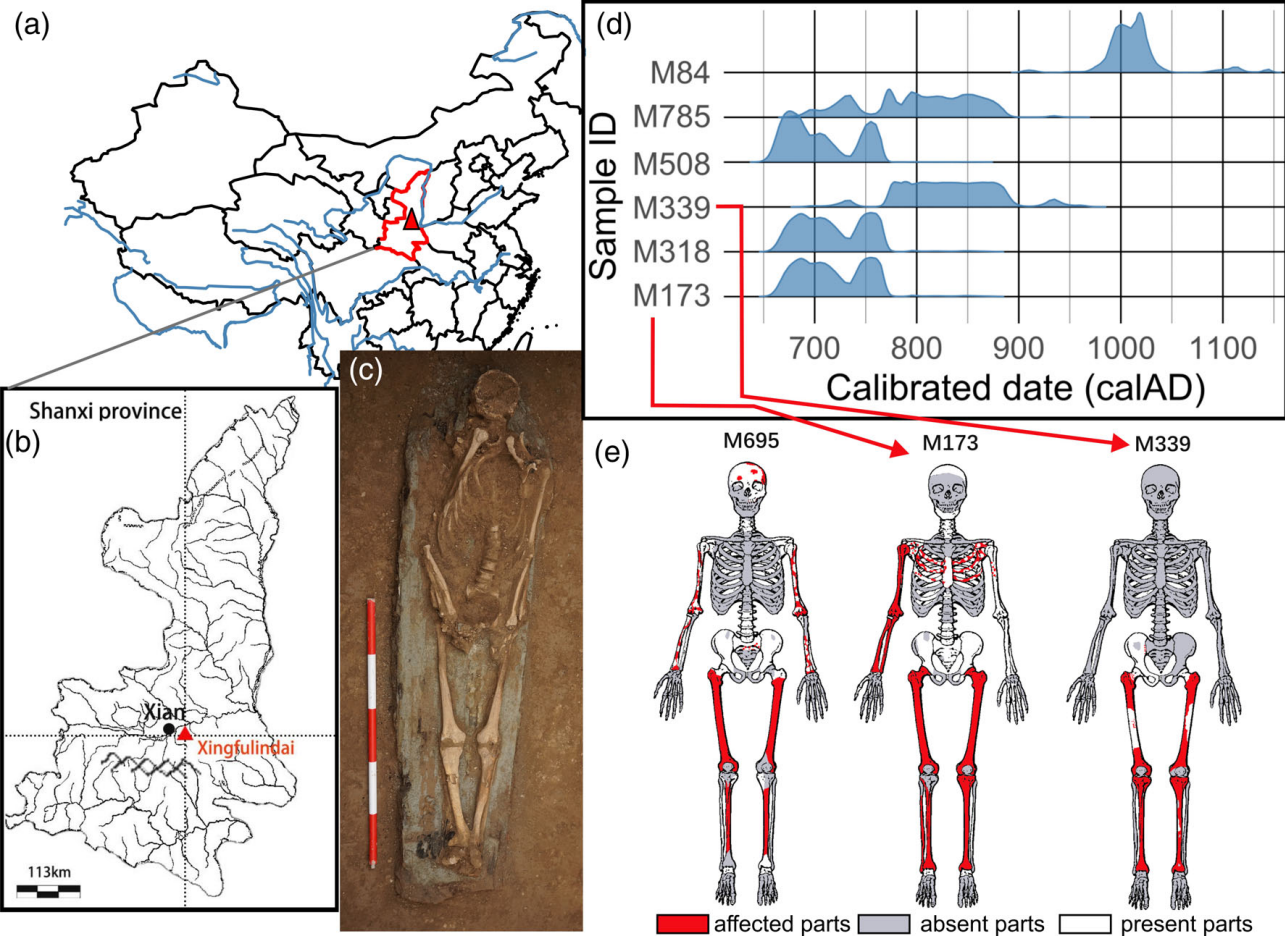
Archeological context of Xingfulindai skeletal materials: (a,b) Location of the Xingfulindai burial area (1090 0′ 37.51″ E, 340 16′ 1.83″ N) in Shaanxi province, China; (c) Burial M173; (d) Distribution of calibrated radiocarbon from Xingfulindai skeletons (Table 1); (e) Three adult skeletons from Xingfulindai with widespread bilateral lesions suggestive of systemic pathology. The red shading indicates areas of the skeleton affected by proliferative or destructive lesions; white areas indicate parts of the skeleton that are present, but do not display lesions; the gray shading indicate parts of the skeleton that were lost due to diagenesis

The Xingfulindai (幸福林带) tomb area (109° 0′ 37.51″ E, 34° 16′ 1.83″ N) is located in the suburb of Xi'an City, Shaanxi Province (Figure 2a‐c; Supplementary Figure 1). In August 2017, the Xi'an Cultural Relics Conservation and Archeological Research Institute excavated the site in a salvage effort coordinated with the infrastructure development project. The excavation uncovered a historic cemetery area 5.38 km north to south and approximately 200 m east to west. The cemetery is located immediately east to the ancient city wall of Chang'an (长安), the ancient name of modern Xi'an, which was the capital of Tang dynasty from 618 to 904 AD and one of the largest cities on Earth at this time. The northern part of the cemetery extends as far as the northeastern corner of the Chang'an's city wall, while River Chan (浐河) demarcates the eastern border of the site. Subsequent field seasons uncovered 1076 km^2^ containing more than two thousand historic burials.

According to funerary inscriptions, the people buried at Xingfulindai were residents of Chang'an. The overwhelming majority of burials were A‐shaped funerary chambers (Supplementary Figure 2a) or two‐part burials in a shape of “folding knife” with a passage and a funerary chamber located at an angle to one another (Supplementary Figure 2c). Funerary offerings including coins, ceramic figurines, as well as inscriptions show that all burials were dated to the Tang (AD 618 to 907) and Song (AD 960–1279) dynasties. These dates are corroborated by AMS radiocarbon dates of the excavated skeletons, carried out by the Beta Analytic laboratory, in Miami, Florida (Table 1, Figure 2d).

**TABLE 1:** Details of 14C AMS‐standard delivery dating of bone samples (Bronk Ramsey, 2009; Reimer et al., 2013)

Sample name	Lab code	Sample material	%C	C:N	δ13C ^o^/_oo_	δ15N ^o^/_oo_	14C age (BP)	±	14C age (cal, 95.4% range)
M84	Beta ‐ 535,813	Bone collagen	40.31	3.2	−18.6	10.2	1020	30	968–1147 cal AD
M173^a^	Beta ‐ 564,644	Bone collagen	30.37	3.4	−15.9	10.7	1290	30	664–770 cal AD
M318	Beta ‐ 535,814	Bone collagen	36.22	3.2	−15.8	10.0	1290	30	664–770 cal AD
M785	Beta ‐ 535,815	Bone collagen	34.51	3.3	−16.8	9.6	1220	30	692–887 cal AD
M508	Beta ‐ 535,816	Bone collagen	40.65	3.2	−13.6	8.6	1310	30	656–727 cal AD
M339^a^	Beta ‐ 535,817	Bone collagen	40.50	3.2	−18.4	9.5	1190	30	722–945 cal AD

a Skeletons with pathological changes suggestive or diagnostic of treponemal disease.

All burials included in this study were normative Tang and Song dynasty burials (Supplementary Figure 2a‐c). M339 was an A‐shaped funerary chamber, 3.5 m long and 2.0–1.7 m wide, oriented north, with the body placed on an earthen platform. Grave goods were placed in the eastern part of the funerary chamber and included ceramic jars, plates, and multiple cattle bones. Burials M173 and M695 were also oriented north, similarly shaped as a “folding knife.” The passage of M173 was 2.7 m long, 0.8–1.4 m wide, and 1.8 m deep; the funerary chamber was 2.4 m long, 1.3–1.9 m wide, and 0.8 m deep. The only offering in the chamber was an undecorated ceramic pot. Similarly, M695 had a passage 2.64 m long, 0.8–0.8 m wide, and up to 1.1 m in depth. The funerary chamber was 2.4 m long, 0.7–1.2 m wide, and 1.1–1.3 m deep. Grave goods included tower jars and copper coins, which date this burial to the middle of Tang dynasty.

The excavations uncovered 1598 individual skeletons (Supplementary Table 1). The biological sex of individuals was estimated by standard methods based on cranial and pelvic morphology, with pelvic morphology given preference (Buikstra & Ubelaker, 1994; Walrath et al., 2004). Age at death for adult skeletons was estimated based on morphological changes in the pubic symphysis, auricular surface, and the sternal ends of ribs, and adjustments proposed for East Asian populations (Zhang, 1982; Zhang, 1986; Zhu, 2014). Based on these methods, 395 skeletons were identified as adult males, 357 as adult females, and 381 as adults of indeterminate sex due to their poor state of preservation. The remaining 465 individuals had unfused epiphyses of long bones and were thus classified as juveniles of unknown sex.

### 2.2 Diagnosing treponemal disease

Pathognomonic lesions due to treponemal disease are only observed when the disease progresses to its advanced tertiary stage (Harper et al., 2011). The nature of the changes observed in bone is a combination of proliferative and destructive lesions widespread throughout the skeleton (Ortner, 2003). Crucial to the development of diagnostic criteria in paleopathology is Hackett's work (1951, 1975, 1976), based on examination of skeletons of people clinically diagnosed with different forms of treponemal disease, and is the only primary paleopathological source on the morphology of bony lesions caused by this disease.

Granulomatous lesions, characterized by a center of necrotic tissue, typical of tertiary syphilis, form within proliferative subperiosteal bone, causing superficial cavitation (Hackett, 1976: 362–396; 429–433). Calvarial *caries sicca*, representing a mosaic of smooth bony nodules, radial scars, and superficial depressions on the outer table of the skull caused by healing and remodeling of superficial necrotic lesions, has been recognized as pathognomonic of treponemal disease since the end of the 19th century (Hackett, 1951, 1975, 1976). In addition, bilateral osteitis and subperiosteal expansion associated with focal cavitation of the long bones are pathognomonic of treponemal disease (Hackett, 1975). Pseudo‐bowing of the tibia caused by enlargement of the diaphysis and periosteal deposits of new bone on the anterior‐medial aspect of the tibia, known as saber shin, has been recorded as strongly suggestive of treponemal disease (Hackett, 1976: 101). Pseudo‐bowing is differentiated from true bowing because there is no distortion of the long axis which more typically occurs in adult treponematosis; true tibial bowing is consistent with congenital syphilis and abnormal growth stimulated by the disease (Hackett, 1976: 100–101; Ortner, 2003: 294).

Hackett (1976) and Steinbock (1976) found that bejel, yaws, and syphilis can cause similar skeletal lesions and are similarly distributed throughout the skeleton. Gummatous destruction (including *caries sicca* of the skull), rhinopharyngitis mutilans (gangosa) and saber shin, as well as generalized subperiosteal proliferative lesions, substantial sclerosis and osteitis, and involvement of the medullary cavity of the long bones have all been recorded in patients suffering from yaws, bejel, and syphilis (Grin, 1952; Hackett, 1976: 433; see review by Baker et al., 2020). These changes tend to be more or less bilaterally symmetrical and affect multiple bones on both sides of the skeleton (Baughn & Musher, 2005; Resnick & Niwayama, 1995; Salazar et al., 2002; Tampa et al., 2014). The congenital form of treponemal disease tends to be linked to the presence of venereal syphilis, which readily penetrates the placental barrier; however, cases of vertical transmission of yaws and bejel have also been documented (Baker et al., 2020; Lukehart & Giacani, 2014; Román & Román, 1986). Hutchinson's incisors and Moon's molars dental malformations are diagnostic of congenital syphilis in infants and children (Hillson et al., 1998; Ortner, 2003); however other bone lesions are related to age and indistinguishable from other forms of treponemal disease (Baker et al., 2020). Lukehart and Giacani (2014): 554) summarize cases of cardiovascular and neurological involvement in patients with yaws and genital lesions in patients with bejel, as well as cases of miscarriages consistent with transplacental transmission in mothers suffering from bejel. Finding evidence for treponemal disease in prepubescent juveniles does not exclude a diagnosis of venereal syphilis, as it can also be transmitted non‐sexually (Krivatkin & Krivatkin, 1997; Stokes et al., 1994). Therefore, distinguishing between different types of human treponemal disease may not be possible based on macroscopic and radiographic observation of individual skeletons (Baker et al., 2020; Mays et al., 2003).

Powell and Cook (2005) and later Harper et al. (2011) developed a scoring system to distinguish lesions as consistent, suggestive of, and specific to treponemal disease. More recently, Baker et al. (2020) Table 1) reviewed the changes caused in the skeleton by treponemal disease and developed a non‐ordinal system to categorize lesions as “consistent with”, “strongly suggestive of”, and “pathognomonic of” treponemal infection. In this study, we use these standard criteria to diagnose possible *Treponema* cases. These categories were assigned based on Hackett (1951, 1975, 1976), who described osseous changes in skeletons of patients diagnosed with treponemal disease during their lifetime. Changes in the postcranial skeleton were deemed by Hackett as “on trial” as they are not specific of treponematosis, but systematic and standardized recording aids with a diagnosis and can help understand the distribution of treponemal disease in the past (Baker et al., 2020: 20). A recent study conducted by Vlok et al. (2020) on Neolithic individuals from Southeast Asia, has also followed a similar criterion for scoring and classification.

The differential diagnosis of proliferative lesions on long bones incudes osteomyelitis, Paget's disease, leprosy, neoplasms, chronic leg ulcers, and metabolic conditions known to cause subperiosteal new bone and destructive lesions, such as fluorosis (Baker et al., 2020; Hackett, 1975). Because the presence of superficial cavitation is pathognomonic for treponemal disease, distinguishing lytic lesions from postmortem pseudopathological destruction was of crucial importance for our analysis. We examined each destructive lesion with a Dino Lite digital microscope at 50× magnification. Any discoloration around the lesion margin, sudden or superficial color change, and/or the presence of a jagged margin were accepted as indicative of postmortem damage, because these features tend to be suggestive of pseudopathology, whereas signs of osteoblastic activity validate bone alterations as true lesions (Buikstra & Ubelaker, 1994; Ubelaker, 1997). Evidence for hypervascularity around the margin and for remodeling inside the foci of destruction were accepted in support of the antemortem nature of destructive lesions.

## 3 RESULTS

Eleven adult individuals from the Xingfulindai site (M84, M695, M173, M339, M114, M26, M508, M682, M785, M810, and M318) had cranial hyperostosis or widespread periostosis or osteitis on multiple long bones suggestive of systemic pathology (Supplementary Table 2). Three of these individuals displayed a combination of lesion patterns suggestive of treponemal infection (Figure 2e). In addition, the infant from burial M888 had widespread *serpens endocrania symmetrica* (SES) on the occipital and parietal bones (Supplementary Figure 3a–d), but no other associated pathology that would allow for a differential diagnosis.

### 3.1 Xingfulindai burial M695

**FIGURE 3:**
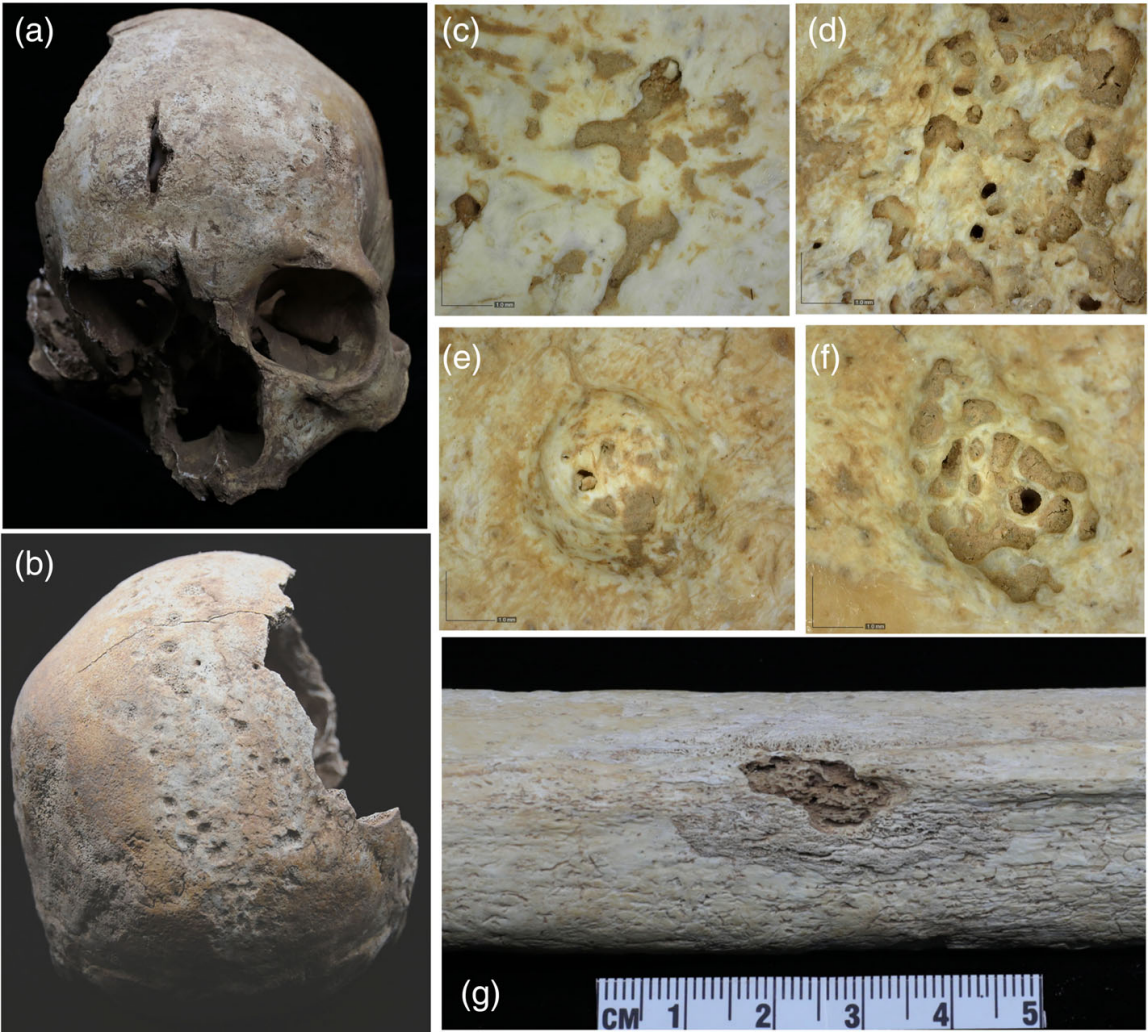
Bone pathology on the skeleton from burial M694: (a) Anterior view of the skull; (b) Posterior view of the skull; (c,d) superficial cavitation on the frontal bone at 50× magnification; (e,f) Superficial cavitation on the parietal bone at 50× magnification; (g) Destructive lesion with reactive margin on the posterior aspect of the left femur

The skeleton of this individual is 75% complete; a partial skull and the majority of the postcranial bones are present (Figure 2e) with many bones sustained superficial postmortem damage. Based on pelvic and cranial morphology, the skeleton belonged to a female, with an estimated age at death of 50 years+. The calvarial bones, including the frontal bone, both parietals, and the occipital bone, present a confluence of superficial shallow destructive lesions consistent with *caries sicca* series described by Hackett (1975). On the frontal bone (Figure 3a,c,d), lesion morphology is consistent with serpiginous cavitation as described by Hackett (1976: 50), while destructive lesions located on the occipital and parietal bones are predominantly circumferential and well circumscribed, with remodeled rims and evidence for a sclerotic reaction. Other lesions showed advanced or partial healing, appearing as thickened shallow depressions (Figure 3b,e,f), consistent with nodular cavitation (Hackett, 1975, 1976). Serpiginous and nodular cavitation are parts of the contiguous series of *caries sicca* progression; hence they are pathognomonic of treponemal infection. A CT‐scan (Supplementary Figure 4) shows generalized thickening of the calvaria and variable density of the diplöe, as well as multifocal ectocranial destruction. The inner table has some small perforations, but otherwise is largely unaffected on the parietal and occipital bones.

The postcranial bones are well‐represented, with only some of the small bones missing. Unfortunately, all bones sustained superficial postmortem damage, complicating their paleopathological evaluation. Both femora and tibiae show circumferential expansion of the diaphysis, and poor preservation of the bone surface, with multiple shallow lytic lesions on the surface. The appearance is a smooth and compact surface accompanied by multiple lytic lesions or cavitations with a reactive margin (Figure 3g), resembling that of gummatous periostosis. Fine pitting and striation are also visible in some areas (Figure 3g).

### 3.2 Xingfulindai burial M173

**FIGURE 4:**
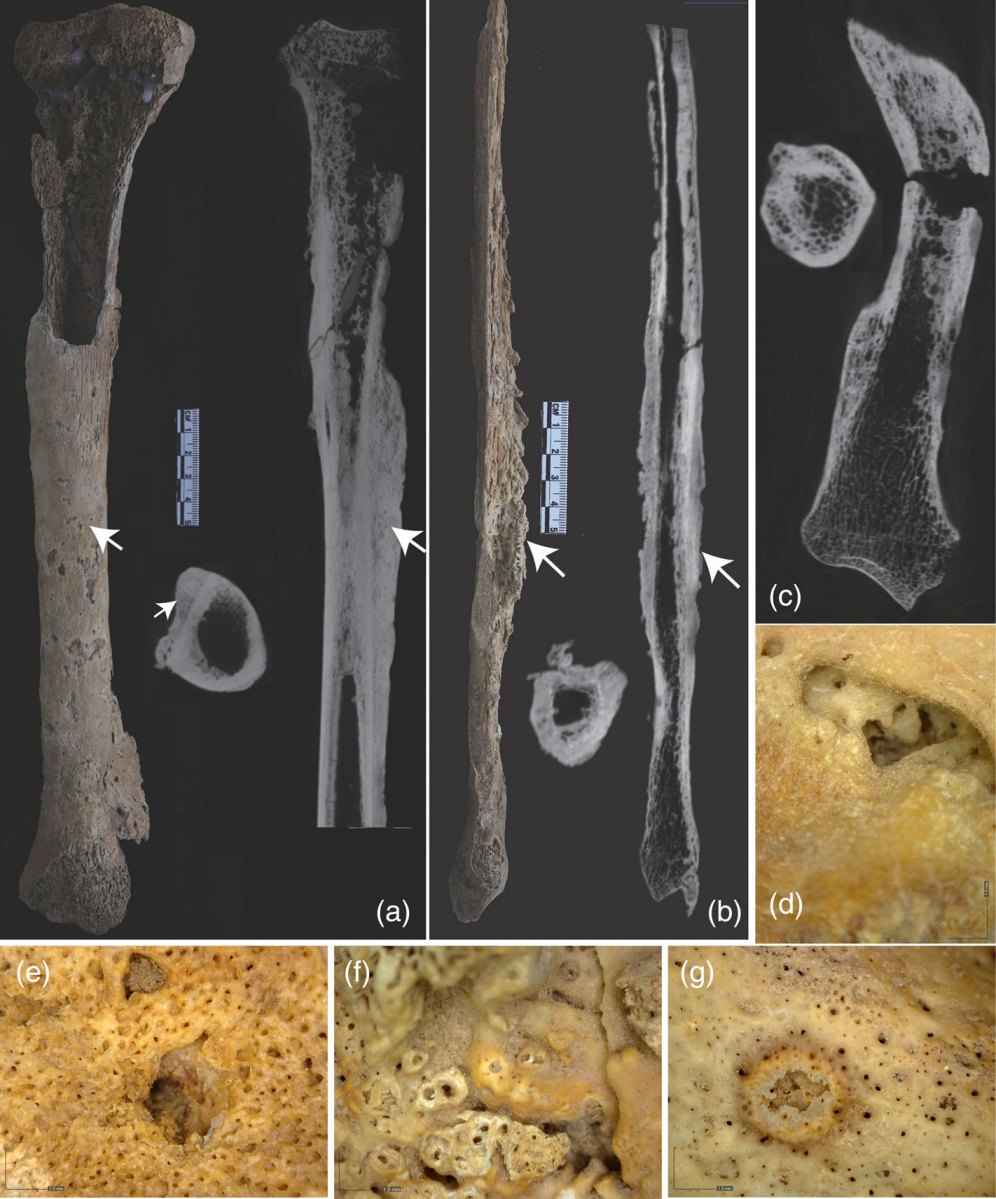
Pathological changes on the male skeleton from Xingfulindai burial M173: (a) The right tibia and CT slices of the right tibia, notice the visibility of original cortex and endosteal bone formation; (b) Florid periosteal reaction on the right fibula and CT slices of right fibula; (c) CT image of the right radius, (d) Superficial lesion on the right radius at 50× magnification; (e–g) Superficial lesion on the right tibia at 50× magnification

The skeleton from M173 belonged to a male with an estimated age at death between approximately 35 and 45 years. Although the skull was mostly destroyed and is represented only as small fragments, preservation of the postcranial skeleton was moderate to good. Extensive pathological changes were observed on all leg bones as well as on the right radius, ulna, and humerus (Figure 4, Supplementary Figures 5–8).

The tibiae were affected by severe osteitis (Figure 4a), although its development was stronger on the right tibia than on the left one. The right fibula displayed evidence for a florid subperiosteal reaction along the shaft (Figure 4b, Supplementary Figure 5). Reactive endosteal bone formation was seen on the CT scans of both tibiae, right fibula, right radius, and distal femora (Figure 4a–c, Supplementary Figures 5–8). Several superficial antemortem destructive lesions were identified on the tibiae and right radius in association with marginal bone remodeling and hyperporosity (Figure 4d–f), although postmortem cortical erosion precluded the possibility of evaluating some destructive lesions on the tibiae. The right radius also displayed severe osteitis and superficial cavitation (Figure 4c,d), but the contralateral radius was not affected. These changes are pathognomonic of treponemal infection (Hackett, 1951, 1975, 1976, Supplementary Table 3).

### 3.3 Xingfulindai burial M339

**FIGURE 5:**
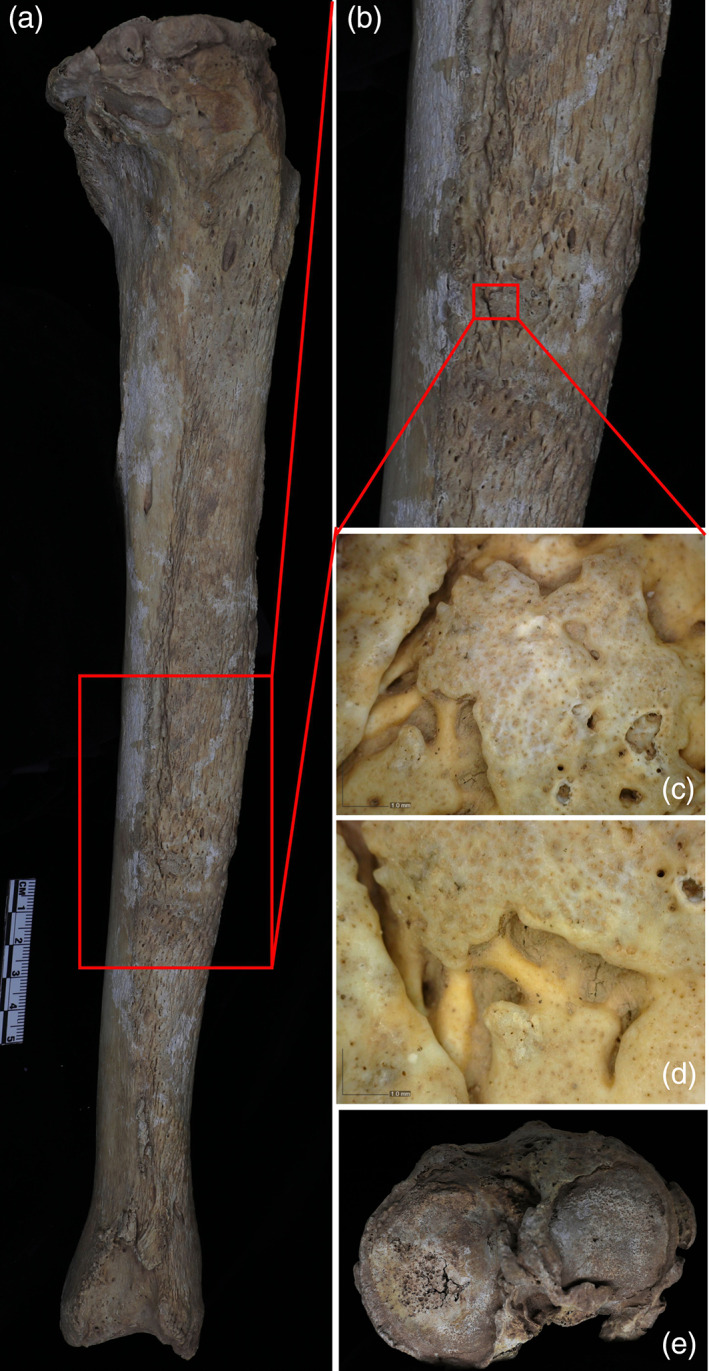
Pathological changes on the male skeleton from Xingfulindai burial M339: (a) Periosteal reaction along the shaft of left tibia; (b) Close up to the periosteal bone proliferation; (c,d) Superficial lesions at 50× magnification; (e) Degenerative changes in the proximal epiphysis (also see Supplementary Figure 9)

Only the inferior portion of the skeleton from burial M339 was preserved (Figure 2e). This skeleton represents a 35–45 year old male based on the fragmentary os coxa. The tibiae show lesions characteristic of diffuse non‐gummatous periostosis, with thickening of the periosteum and the cortex, and with dense sclerotic bone (Figure 5, Supplementary Figure 9). This apposition results in an irregular, rugose, and dense surface with slight pitting and fine striation affecting the entirety of the shaft. Two superficial destructive lesions were observed on the lateral aspect of the tibiae, consistent with gummatous periostosis (Figure 5c,d). Both knee joints show evidence for eburnation and erosion (Figure 5e, Supplementary Figure 9b,c), consistent with syphilis (Hackett, 1976: 106). Anteromedial deposition of new bone results in a saber‐shaped tibia. Additionally, there is moderate bilateral enthesophyte formation on both tibiae along the interosseous crests and popliteal lines. There are severe spiculated enthesophytes at the attachment site for the semimembranosus muscle and at the tibial tuberosity of the right tibia. Severe marginal osteophyte formation is present in both knee joints, as well as eburnation coupled with lytic destruction of the joint surface.

### 3.4 Differential diagnosis

Following Harper et al. (2011) and Baker et al. (2020), Supplementary Table 3 summarizes the findings from our results and categorizes them as pathognomonic (P), strongly suggestive (S), and consistent (C) with a set of diagnoses. The skeleton from burial M695 exhibits advanced *caries sicca* and bilateral gummatous periostitis, both of which are pathognomonic for treponematosis. The presence of the subperiosteal reaction and superficial cavitation observed on the leg bones, which are pathognomonic of treponemal infection reinforce the diagnosis. The differential diagnosis for this skeleton includes a secondary malignancy ‐ metastatic carcinoma (Supplementary Table 4). Although some metastatic tumors, such as breast and prostate cancers, can present as a combination of both destructive and proliferative tumors, these malignancies are aggressive and should not present evidence for long‐term remodeling characteristic of a chronic condition, as seen on the skeleton of M695. These observed changes are inconsistent with metastasizing neoplasms, as those lesions are rarely limited to the outer table of the bone and they rarely present well remodeled margins (Ragsdale et al., 2018).

Postmortem alterations to the skulls of M173 and M339 complicate diagnosis. Diffuse osteitis, periostosis, and endosteal bone proliferation, combined with several small superficial destructive lesions, are strongly suggestive of treponemal infection. The lesions found on the remains from M173 were more severe on the right arm and the left leg bones. Their presentation is typical of a slow progressing and chronic systemic pathology and, therefore, excludes diagnosis of an overwhelming majority of the conditions listed in Supplementary Table 4. Although osteomyelitis infection can also trigger endosteal bone proliferation and florid periosteal reaction, it is generally associated with pus buildup leading to cloacae formation (Ortner, 2003) not seen in M173. Having multiple bones affected on both sides of the body is also not typical for osteomyelitis. Although the florid periosteal reaction as well as excessive ossification in articular and entheseal areas similar to those seen in M173 can be triggered by fluorosis, this would not explain the osteitis superficial destructive lesions and endosteal bone formation (Brickley & Ives, 2008). A complete absence of exostosis and enthesophytes on the vertebral column is also inconsistent with fluorosis.

The lesions observed on the skeleton of M339 are consistent with and strongly suggestive of treponemal disease, but do not rise to the level of being pathognomonic. In the absence of multiple gummatous destructions, the diffuse periostosis observed on the leg bones is also consistent with fluorosis and a number of pathological processes that can trigger systemic inflammation (Ortner, 2003) (Supplementary Table 4). Thus, using the scoring system developed by Harper et al. (2011, Table 2), M695 would score 5 out of 5, M173 receives scores between 3 and 4, while M339 would score 2 to 3.

## 4 DISCUSSION

The present study, documenting the presence of treponemal disease in north‐central China between AD 656 and 1147, challenges the da Gama model that syphilis spread to East Asia. Even though the skeletal signatures of the disease do not allow us to conclusively distinguish between ST syphilis, yaws, and bejel (Buckley & Dias, 2002), the ecological settings at Xingfulindai were not typical for yaws and bejel ecology, because these strains only occur in the tropics, or in hot, dry environments respectively.

The part of China where Xingfulindai is located is characterized by continental semi‐arid climate with warm and humid summers and cold dry winters, during which the temperatures frequently dip below freezing. Even though the climate in China during the Tang dynasty was characterized by slightly warmer average annual temperatures, the northeastern territories of China experienced cooling, particularly during the winters (Yan et al., 2015). Furthermore, the urban setting of the Xingfulindai site in association with the Tang capital of Chang'an, one of the largest and ethnically diverse cities of its time as well as the absence of lesions on pre‐pubescent skeletons favors the diagnosis of ST syphilis over yaws and bejel. Of course, given the evolutionary plasticity of *Treponema*, it is possible and even likely that now extinct strands of this pathogen existed in the past. Indeed, *Treponema pallidum* genome of a previously unknown strain from early modern Europe has been identified in a recent study (Majander et al., 2020). Ecology, clinical manifestations, and modes of transmission of infectious diseases caused by those extinct strains may not correspond precisely to the modern clinically documented cases, as was considered previously for the North American cases by Baker (2005), Hutchinson et al. (2005) and Cook (2005). We cannot dismiss such a possibility for the early cases of treponemal disease in China.

### 4.1 Literary evidence of ST syphilis in early China

Consistent with the Vasco da Gama model, the earliest direct description of ST syphilis can be found in “Compendium of Materia Medica” 本草纲目 (c1578‐1596) by Li Shizhen 李時珍 (Li, 2016 [1596]), which is illustrative of the cultural perception and treatment of ST syphilis in historic China. Li described a sexually transmitted disease, which he referred to as “杨梅疮” or myrica. He named it after the plant *Myrica rubra* (bayberry), which produces bright red bumpy fruits reminiscent of syphilis sores. He noted that the disease started spreading from the Lingbiao area of Guangzhou. His text also described a treatment for the disease, including the use of mercury ointment:

“杨梅疮，古方不载，亦无病者，近时起于岭表，传及四方，盖岭表风土卑炎，岚瘴熏蒸，饮啖辛热，男女淫猥。湿热之邪积蓄既深，发为毒疮。遂致互相传染，然皆淫邪之人病之。“In the past, no books mentioned syphilis (myrica sores) nor how to cure it. Syphilis probably started in the Guangxi and Guangdong area and began to spread. The fields there are low‐lying and the weather is hot, humid, and foggy. Thus, people there like to eat spicy food. Because of this, it makes symptoms worse, causing toxic sores which begins to infect others, but only infects those who had sexual behavior.”

医用轻粉，银朱劫剂，五、七日即愈。其性燥烈，善逐痰涎。涎乃脾之液，此物入胃，气归阳明，故涎被劫，随火上升，从喉颊齿缝而出，故疮即干痿而愈。若服之过剂，及用不得法，则毒气窜入经络筋骨之间，莫之能出。痰涎既去，血液耗涸，筋失所养，营卫不从，变为筋骨挛痛，发为痈毒疳漏。久则生虫为癣，手足皱裂，遂成废痼。“Use medical calomel (mercury chloride) or cinnabar (mercury sulfide) to cure it. It usually takes five to seven days to cure. These types of drugs are so strong that they will easily make the spleen produce saliva. When the drugs go into the stomach, it will produce good energy and send it to yangming. Then, the energy will go up with the saliva that is produced from the spleen via the throat to the mouth, and it will gradually make the sores smaller, eventually curing them. However, if the dose is too large, then it can worsen the symptoms because the overdose can be toxic and produce bad energy to go into the meridian system. What's even worse, the bad energy has no way to get out, which will result in having less saliva, low blood pressure, weak bones, malnutrition, and then causes bone pain. This ultimately results in ulcer and gum disease. If the patient ignores this for a while, it will cause skin ulcers, and their hands and feet will begin cracking and develop a chronic illness.”

其类有数种，治则一也。其证多属厥阴，阳明二经，而兼乎他经。邪之所在，则先发出，如兼少阴太阴，则发于咽喉。兼太阳、少阳，则发于头耳之类。 “Although there are many types of syphilis, the principles are the same. Syphilis will send bad energy to jueyin and yangming. People will feel discomfort in different parts of their body, depending on where the bad energy moves to. For example, if the energy goes to shaoyin and taiyin, the patient will feel discomfort in their throat. If it moves to taiyang and shaoyang, then the patient will feel discomfort around the head and ears.” In this passage, “jueyin,” “yangming,” “shaoyin,” “shaoyang,” “taiyang,” and “taiyin” are the circulatory tracks of traditional Chinese medicine, which can be thought as the major flows or meridians of the qi energy, with distinct locations in the body (Needham, 2000:48). For instance, “jueyin” has the most internal position, “yangming” is anterior, “taiyang” is the most external, etc. Different circulatory tracts are related to different internal organs (for more details on meridians in traditional Chinese medicine see Shen, 2021).

惟土茯苓气平，味甘而淡，为阳明本药。[…] 今医家有搜风解毒汤治杨梅疮，不犯轻粉。病深者月余，浅者半月而愈。服轻粉而筋骨挛痛，瘫痪不能动履者，服之亦效。其方用土茯苓一两，薏苡仁、金银花、防风、木瓜、木通、白鲜皮各五分，皂荚子四分。气虚，加人参七分；血虚，加当归七分。惟忌茶、肉、法面，房劳。盖秘方也。 “Powdered tu fuling (*Smilax glabra*) is a good remedy to cure yangming disease, because it tastes sweet and light, which helps smooth the “fire”—bad energy. […] Presently, the soufeng jiedu (搜风解毒) soup is used to treat syphilis. Curing patients with serious symptoms by drinking soufeng jiedu will take a month; those with light symptoms are cured within half a month. To make soufeng jiedu soup use one *liang* (equivalent to approximately 37 g in the Ming Dynasty) of 土茯苓 tufuling (*Smilax glabra)* and 1.5 g of 薏苡仁 yiyiren (Job's tears: *Coix lacryma‐jobi*), 金银花 jinyinhua (Japanese honeysuckle *Lonicera japonica*), 防风 fangfeng (*Saposhnikovia divaricate*), 木瓜 mùguā (papaya, *Carica papaya)*, 木通 mutong (chocolate vine *Akebia quinata*), and 白鲜皮 báixiǎnpí (*Dictamnus albus)* each respectively, as well as 1.2 g of 皂荚子 zaojiazi (Acacia). For people who do not have enough good energy, one can add 人参 rencan (Asian ginseng, *Panax ginseng)* 2.1 g; people who have low blood pressure can add 当归 danggui (female ginseng, *Angelica sinensis)* 2.1 g). Cook all of the herbs with two bowls of water and make soup. Drink the soup three times a day and avoid drinking tea, eating beef, lamb, and chicken; maintain abstinence. This is the secret formula.”

The efficacy of soufeng jiedu soup against the infection may have relied on bacteriostatic properties of polyphenols and phytosterols contained at a high concentration in *Smilax glabra* rhizomes (McMurray et al., 2020; Xu et al., 2013), although it is not clear whether sufficient concentration of these compounds was achievable by preparing the soup. Consumption of Smilax sp. root can have a sudorific effect (Jiang & Xu, 2003), which could be perceived as curative against syphilis. The root of *Smilax* sp., also referred to as “China root” was introduced to Europe in 1535 as a miracle remedy for syphilis (Morgan, 1957; Winterbottom, 2014).

Even though Li′s text translated above presents the earliest commonly recognized historic description of syphilis in China, a much earlier description of symptoms consistent with syphilis can be found in a medical text by Sun Simiao from the 7th century AD in Qianjinfang (千金方). The text described two types of sores, *duijing chuang* 妬精瘡 and *yinshi chuang* 陰蝕瘡, but did not associate them with syphilis (Morgan, 1957). However, *dujing* was described as affecting the prepuce in males and the labia in females and was suggested to be treatable by calomel. By the mid‐14th century AD, *dujing* sores were recognized to be linked to unclean intercourse and required mercury as a treatment (Wong & Wu, 1936). Of course, such sores could be caused by other sexually transmitted diseases, such a herpes and cancroid.

### 4.2 Paleopathological cases of treponemal disease in early China

At odds with multiple historic sources, the paleopathological literature from China suggests a much earlier presence of treponemal disease in this part of the world, although the availability of well‐provenanced skeletal collections has been limited until recently. Calvarial *caries sicca* pathognomonic or strongly suggestive of treponematosis has been described on a skull from Fujian Province dated to the Song dynasty (AD 960–1279) (Zhang, 1994) (Figure 1a). However, no postcranial material associated with the skull was available to assess possible long bone involvement. Furthermore, the skull has not been directly dated and may represent a later burial (Zhang, 1994). Two possible Bronze Age cases of treponematosis have been reported based on postcrania from northeastern Qinghai Province, in the vicinity of Xining (Kayue culture) (500 BC–AD 150) (Suzuki et al., 2005) (Figure 1a). In this study, the authors report multiple cases of subperiosteal bone formation on tibiae and one case of bilateral distribution of periosteal lesions with their plaque‐like nodular appearance on both distal femora. Based on these findings, Suzuki et al. (2005) proposed that syphilis was introduced to China via trade routes from the west (Figure 1b, green line). The network of trade routes between East Asia and the rest of the continent can be traced back as far as the Neolithic, when new cereals, including wheat and barley, were introduced to China (Flad et al., 2010). Because the study was carried out on collections excavated during the 1970s, when selective storage of long bones was the norm, precluding evaluation of the full skeleton, the validity of this case remains controversial. Consistent with Suzuki et al.’s (2005) findings, another paleopathological case of treponemal disease has been documented in the same area from the Taojiazhai archeological site (Zhang, 2013) dating to the Han (206 BC–AD 220) and Jin (AD265–420) dynasties, albeit the characteristics of the lesions found there did not reach the level of being diagnostic.

The Island Southeast Asia and Pacific model developed by Buckley and Oxenham (2016), reviews abundant skeletal evidence for treponemal disease, likely yaws, throughout the Pacific, with the earliest case from Micronesia, dated to c AD 800. They note a clear pre‐European presence of treponemal disease in Polynesia, Micronesia (Figure 1b blue line), and insular Southeast Asia, suggesting two possible routes for the spread of infection, either from the west coast of South America, or from the west via the Middle East into insular Southeast Asia, and further on to Micronesia, although the former route of transmission would not explain the earliest cases of the disease in Micronesia. Favoring the latter hypothesis, Vlok et al.’s (2020) work from prehistoric Vietnam has shown the earliest possible evidence for treponemal disease in Southeast Asia, associated with an archeological site dating to 4000 years ago.

### 4.3 Sociocultural context of Xingfulindai cases

Recently, Baker et al. (2020) noted that given the plasticity of *T. pallidum* it is important to examine the environmental and sociocultural conditions that favored the emergence and spread of the new forms of treponemal disease. The Xingfulindai cases of treponemal infection, with direct radiocarbon dates that place skeletons prior to 1400s, and their association with the large urban population of Chang'an, present us with such an opportunity. According to the New Book of Tang (chapter 41.27:1669), the capital city of Chang'an had 362,921 households with a total population of 960,188 people within its limits and ruled over 20 provinces. The population of Chang'an was multiethnic, with a notable influx of people from the northwestern frontier area of imperial China as well as Persia (Da, 2001:10). Feng (2010) analyses of Tang stories of romance depict the vibrant culture of romance in Chang'an capital city during the Tang dynasty, which saw regular influxes of male examinees in pursuit of *jinshi*, the degree needed for elevation to political power, and the complexity of their relations with courtesans of the city. There are many accounts in ancient Chinese writing of sexual relationships and sexual activity (Goldin, 2001; Yao, 2002) and historical evidence for the practice for polygamy with concubines living with married couples, and courtesans being an integral part of society during the Tang dynasty and codified in the Tang law system (Yao, 2002). Tang *Lu Shu Yi*, the set of Tang decrees, defined the number of concubines that officials of different rank could possess, whereby officials of first rank could have ten concubines. Living with a wife and a concubine, while also establishing relations with a courtesan, was a common practice for the wealthy males of the time.

The period contemporaneous with the Xingfulindai burials was also the time when the travel along the network of international trade routes spanning a vast geographic region, later dubbed the Silk Road (2nd century BC–15th century AD), was extremely active, promoting an increased level of interaction, bringing into contact previously isolated areas (Waugh, 2010). As such, not only did this increased interconnectedness facilitate the spread of people, goods, and religions, but the proliferation of pathogens too. Classically, the movement of people along the Silk Road has been associated with the spread of *Yersinia pestis*, or the bubonic plague (Morelli et al., 2010). Furthermore, the Tang dynasty rule was supportive of the influx of people from the outside of the empire and encouraged assimilation. The Huiyao Tang history text by Wang Pu (ca. AD 961) recorded that a Tang decree of AD 628 permitted foreigners to marry local women, although it prohibited foreigners from taking their local wives back to their countries.

If these pathological cases from Xingfulindai represent individuals with sexually transmitted syphilis, it is important to investigate how this would have affected the community by considering the known transmission rate of this disease, and the functional effects on people. Unfortunately, unbiased research on sexual transmission of syphilis is rare (Stoltey & Cohen, 2015). However, the research that has been done shows that the transmission rate is 51%–64% per partnership (Schober et al., 1983). Transmission rates within a population are dependent on the transmission probability per partnership and the rate of acquisition of sexual partners and length of infectiousness of the disease (Garnett, 2008).

Although there are inherent biases in estimating disease prevalence in past populations from skeletal samples (DeWitte & Stojanowski, 2015), we can make cautious estimates. It has been shown in a survey of 2000 cases of active untreated syphilis in Norway from 1890–1920 that only about 1 percent of individuals showed skeletal involvement (Gjestland, 1955). Therefore, we can assume that the number of individuals in a population who will show skeletal evidence for syphilis is small. Given that there is skeletal evidence in two individuals out of 1598, this is slightly more than the rate that has been argued in pre‐antibiotic European populations (Hackett, 1976: 114). If we consider only the adults from these Chinese sites this raises the prevalence to greater than two individuals per 1000. The true prevalence would be significantly higher, considering that many of the skeletons analyzed did not have sufficient skeletal preservation to undertake a pathological examination. Prior to antibiotic treatment, ST syphilis had deleterious effects, with individuals experiencing long‐term suffering, disfigurement, cardiovascular and neurological damage, and mortality (Singh & Romanowski, 1999).

## 5 CONCLUSIONS

The present study documents skeletal cases of advanced treponemal disease directly dated to 664–945 cal AD, excavated from a cemetery in the ancient city of Chang'an, one of the largest urban centers of its time, located in north‐central China. Based on the ecology of treponemal disease, these cases may represent sexually transmitted syphilis, challenging the Columbian/Vasco Da Gama model for the spread of treponemal disease to China. Given recent findings of the zoonotic presence in Africa, it seems likely that different strains of *Treponema pallidum* traveled with human migration across Europe and Asia several thousand years ago and different forms of this disease emerged when the pathogen was introduced to new environments. The effect of this disease would have undoubtedly been devastating for many in the community with an estimated high prevalence, possibly related to the social practices of concubines and courtesans that were central to the social fabric of the Tang dynasty.

## CONFLICT OF INTEREST

The authors declare no potential conflict of interest.

## AUTHOR CONTRIBUTIONS

**Yawei Zhou:** Conceptualization (equal); data curation (lead); formal analysis (equal); funding acquisition (equal); investigation (lead); project administration (equal); resources (equal); supervision (lead); visualization (equal). **Guoshuai Gao:** Data curation (equal); investigation (equal). **Xiangyu Zhang:** Investigation (supporting). **Bo Gao:** Data curation (supporting); investigation (supporting); resources (supporting). **Chenggang Duan:** Data curation (supporting); investigation (supporting). **Hong Zhu:** Formal analysis (supporting); supervision (supporting). **Aida R. Barbera:** Formal analysis (supporting); investigation (supporting); writing – original draft (supporting). **Siân Halcrow:** Conceptualization (equal); funding acquisition (equal); methodology (supporting); writing – original draft (supporting). **Kate Pechenkina:** Conceptualization (lead); formal analysis (lead); investigation (equal); methodology (lead); project administration (equal); resources (supporting); supervision (equal); visualization (equal); writing – original draft (equal).

## Supporting information


**Figure S1** Supplementary figures

**Table S1** Supplementary tables

## Data Availability

The data that supports the findings of this study are available in the supplementary material of this article
