# Supplementary material for: Identifying treponemal disease in early East Asia
Source: Am J Biol Anthropol. 2022 Apr 27;178(3):530–43. doi: 10.1002/ajpa.24526 (PMC9545539; doi:10.1002/ajpa.24526)

Supplementary Fig. 1: Excavations at Xingfulindai archaeological site.

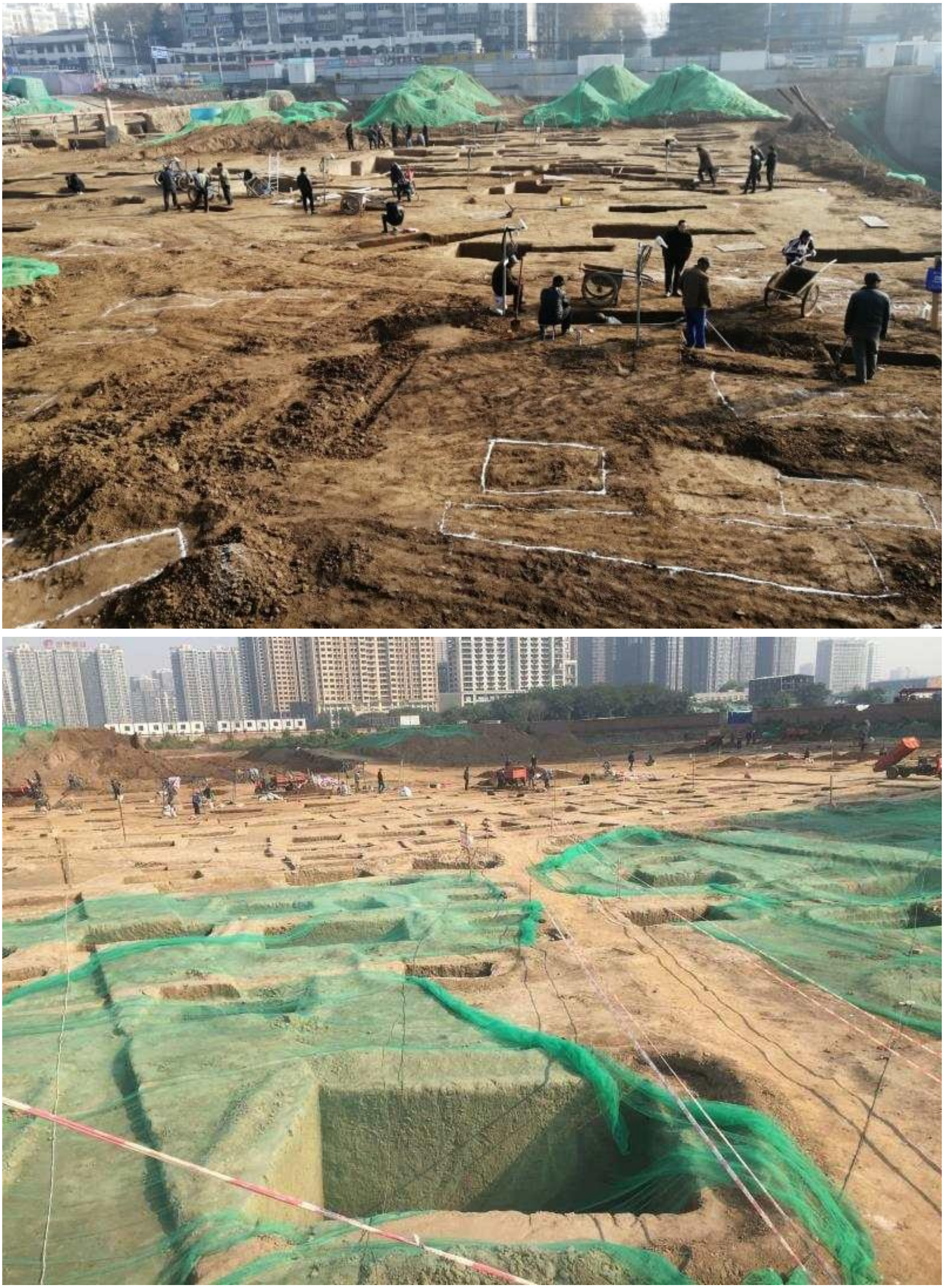

Supplementary Fig. 2: Xingfulindai burials: A - M695, B - M173, C - M339.

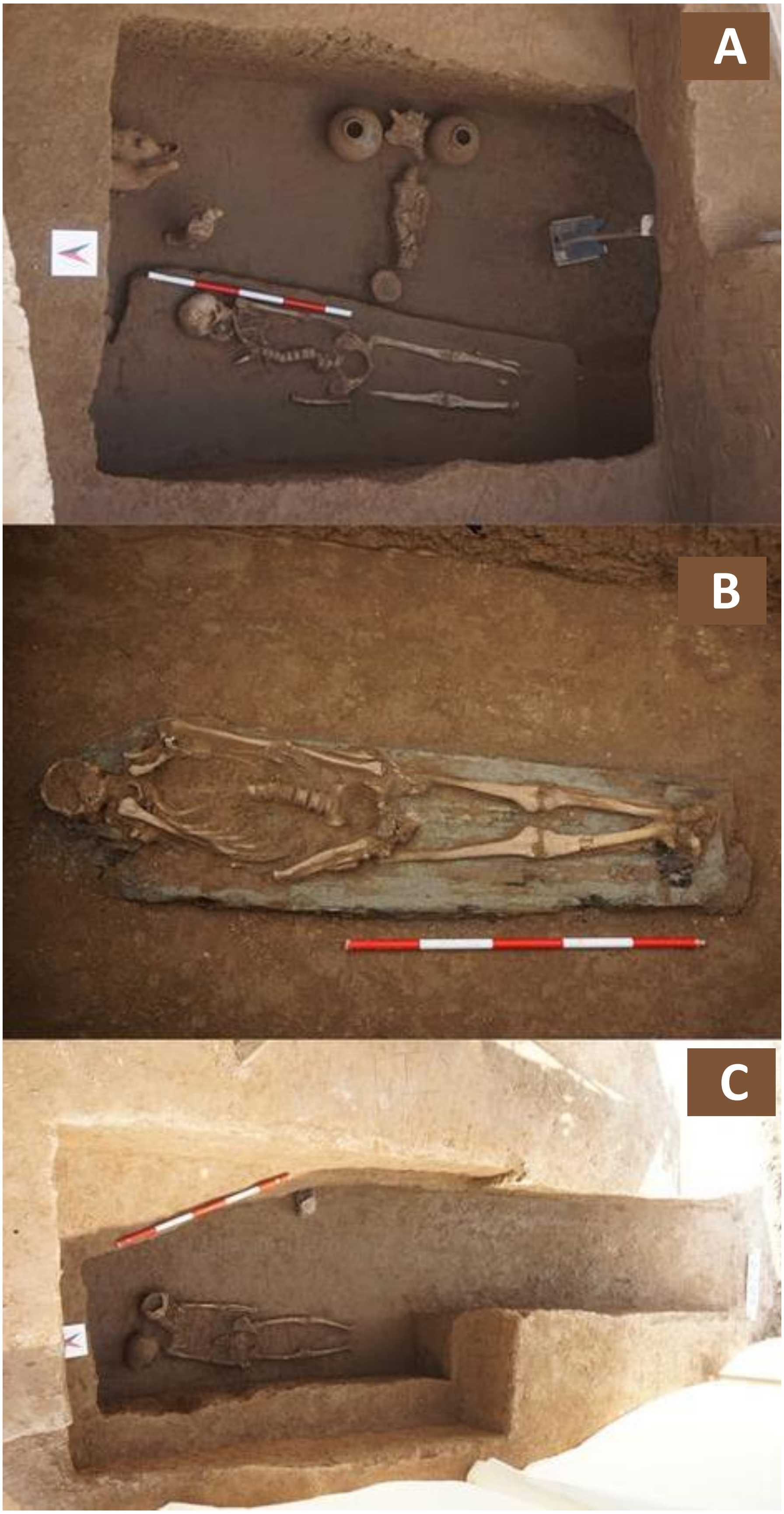

Supplementary Fig. 3: Endocranial pathology on the infant bones from Xingfulindai burial M888.

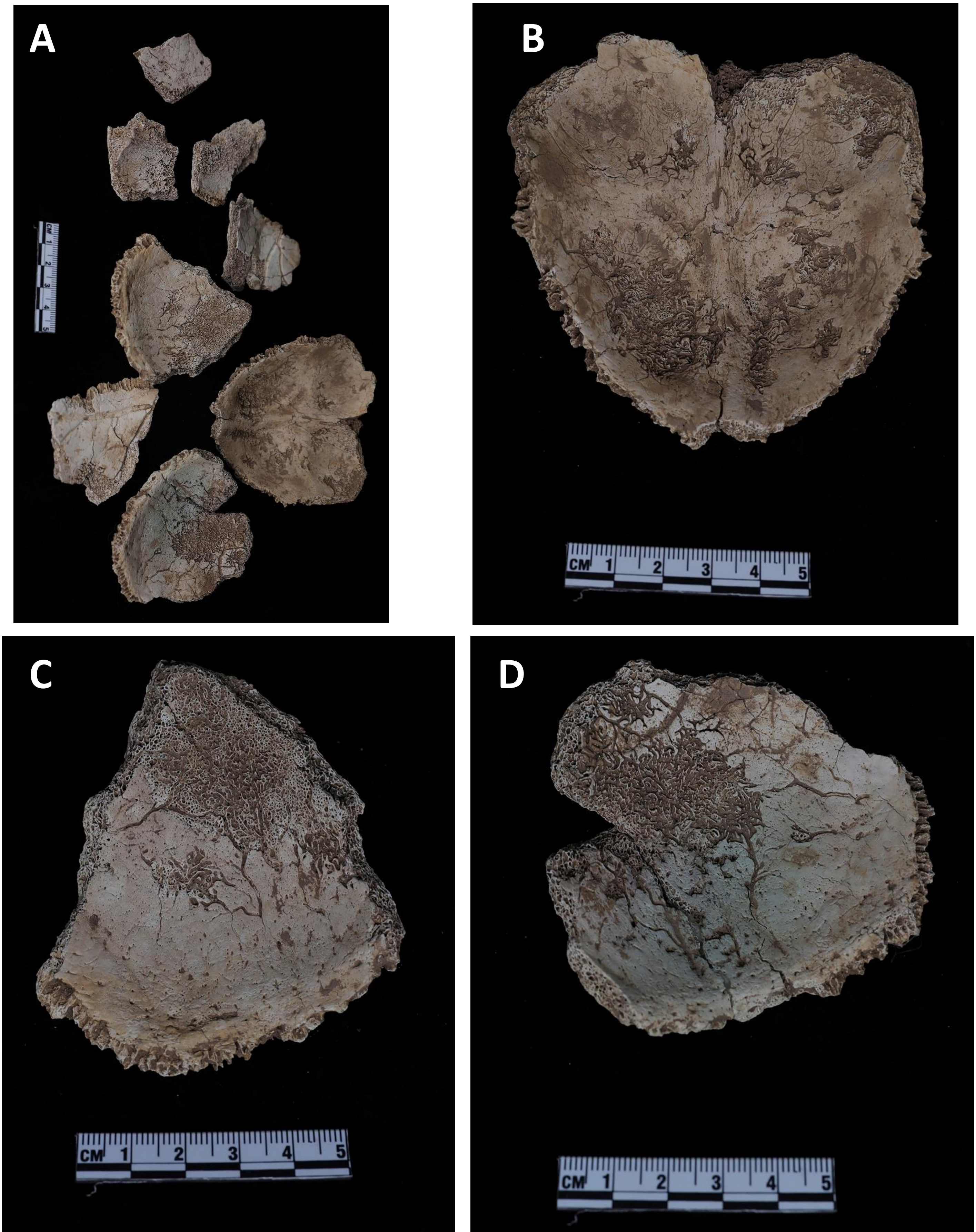

Supplementary Fig. 4: Destructive lesions on the skull from Xingfulindai burial M695.

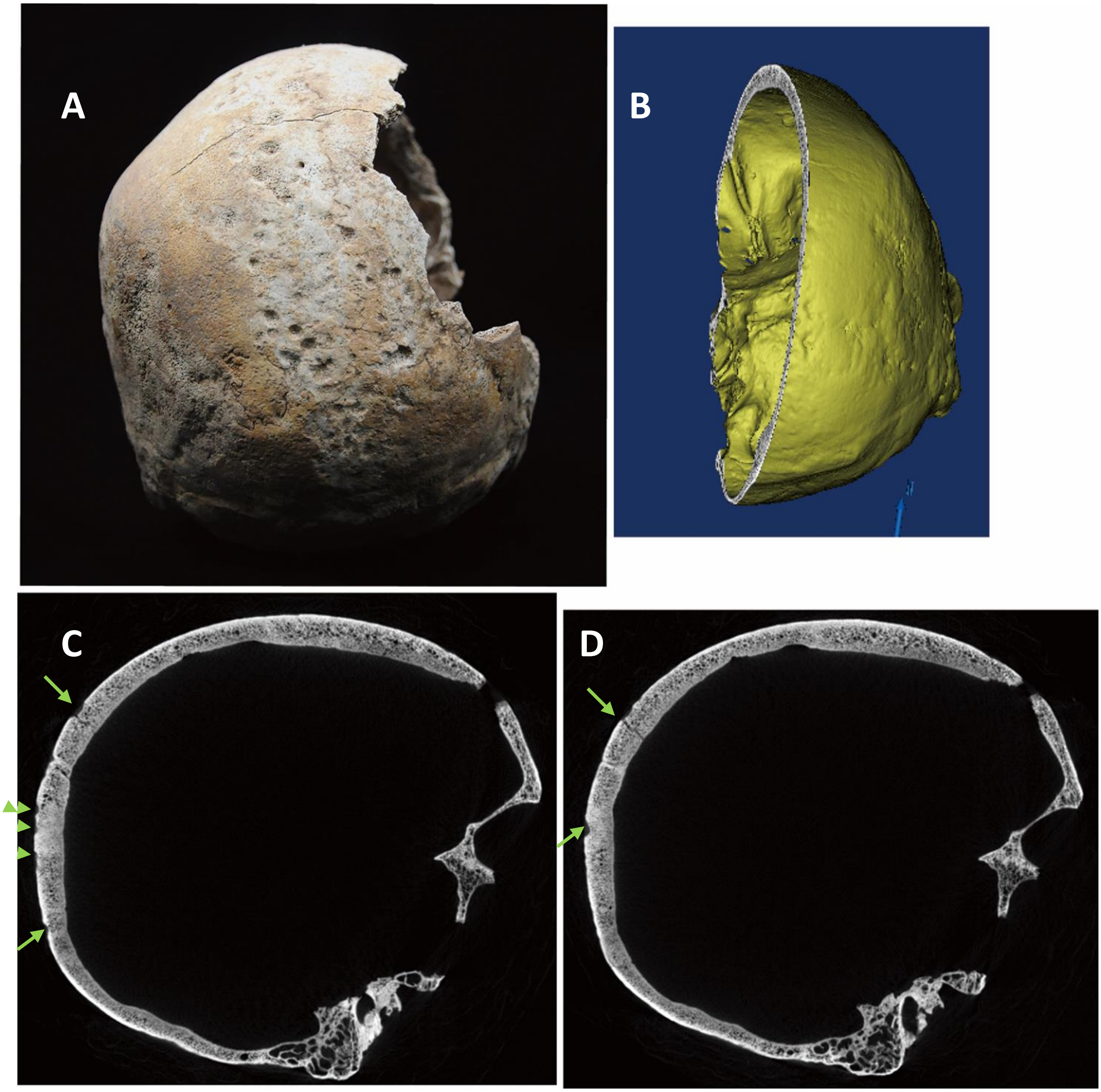

Supplementary Fig. 5: Pathological changes on the right tibia from Xingfulindai M173.

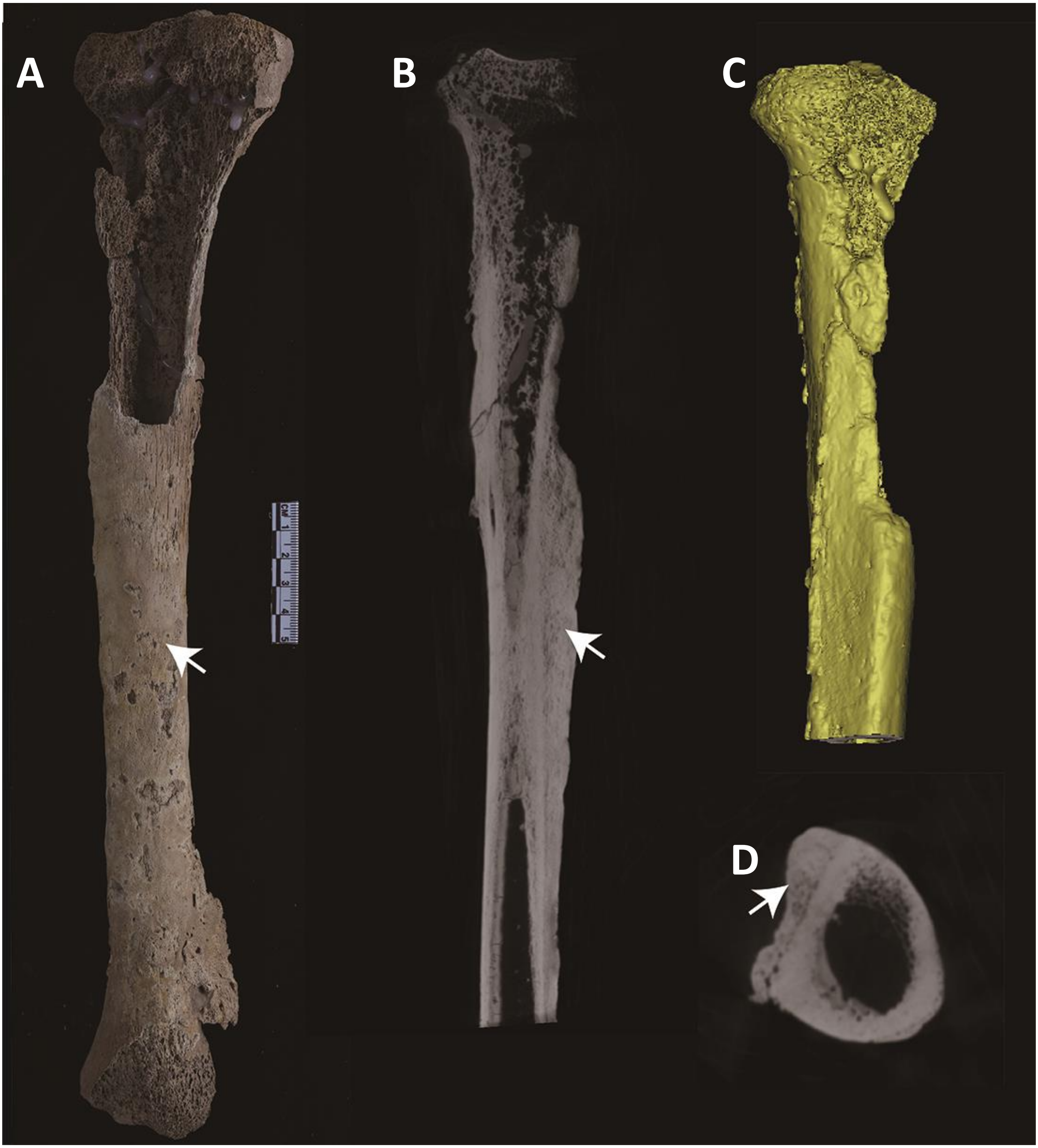

Supplementary Fig. 6: Pathological changes on the right fibula from burial Xingfulindai burial M173.

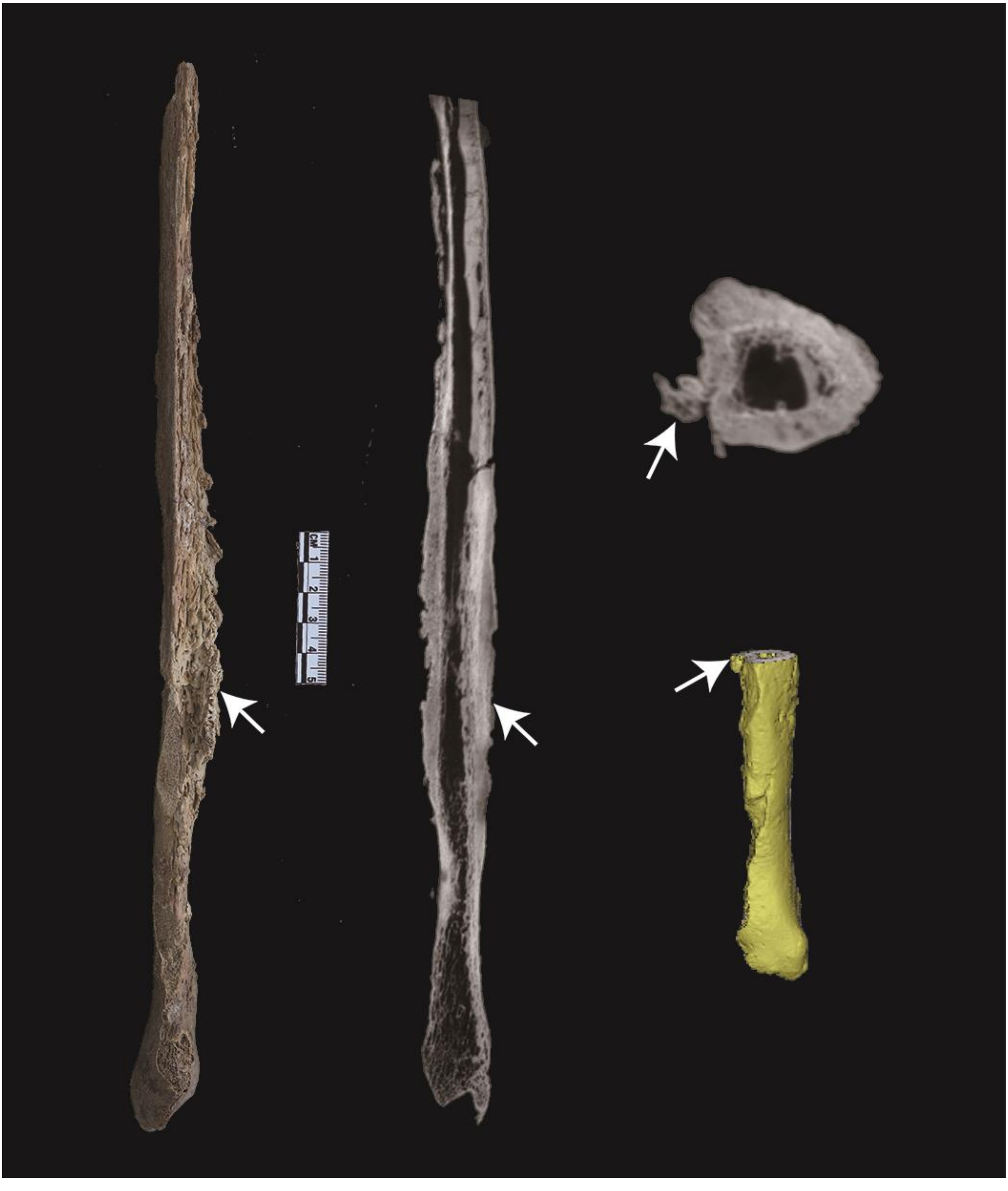

Supplementary Fig. 7: Pathological changes on the right radius from burial Xingfulindai burial M173.

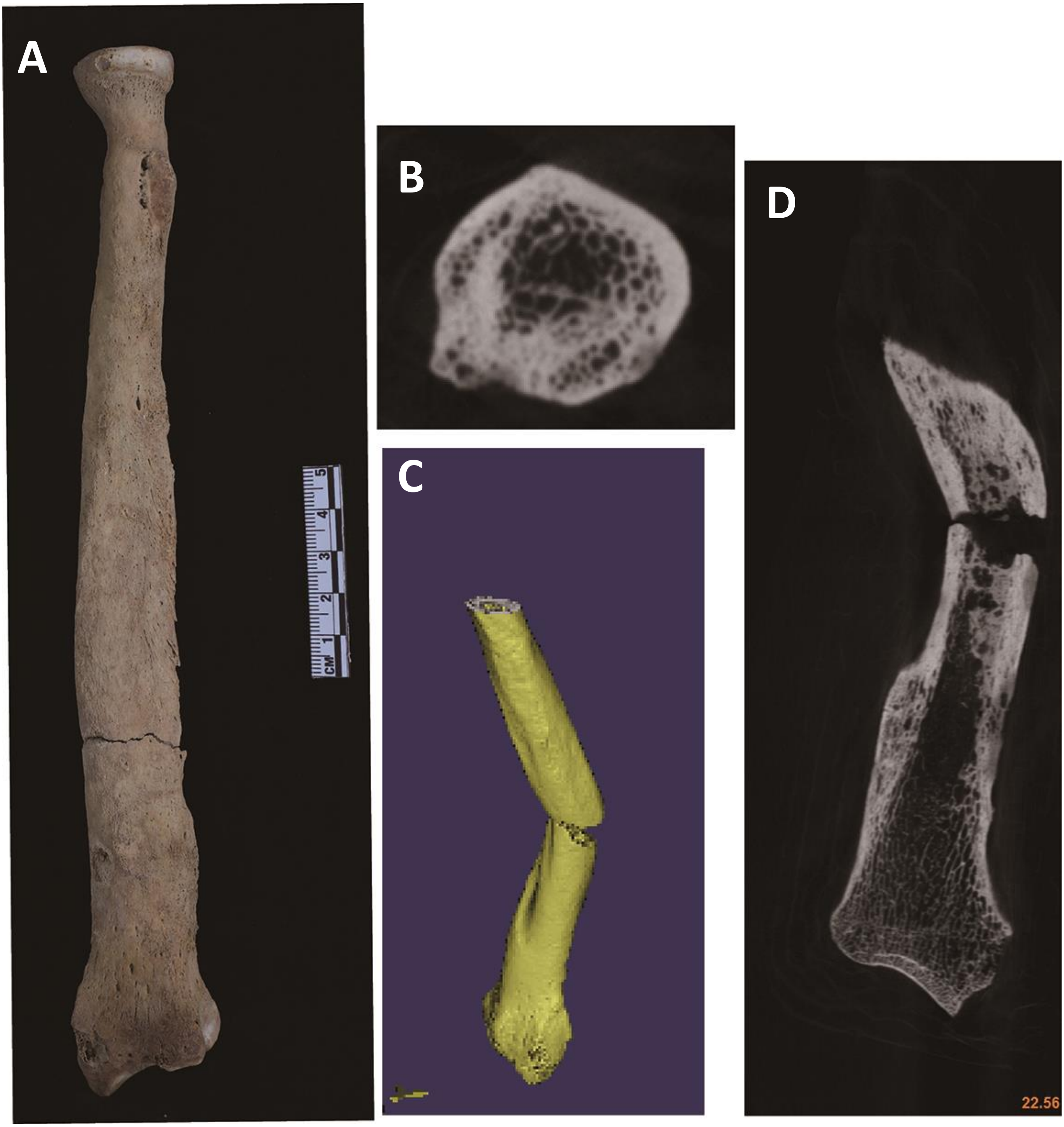

Supplementary Fig. 8: Pathological changes on the right femur from burial Xingfulindai burial M173.

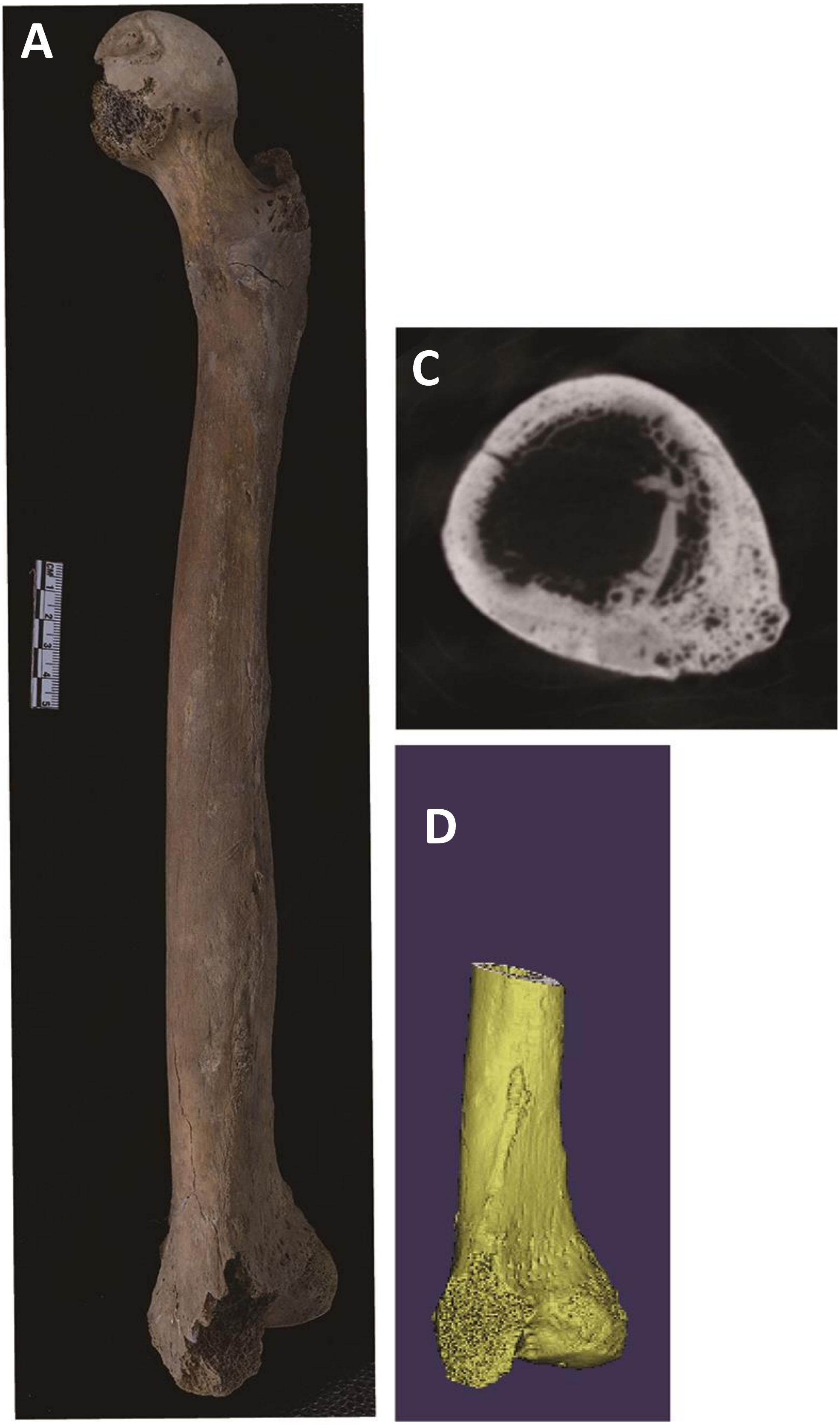

Supplementary Fig. 9: Pathological changes on the left tibia (A-B), right femur (C), and left tibia (D) from burial Xingfulindai burial M339.

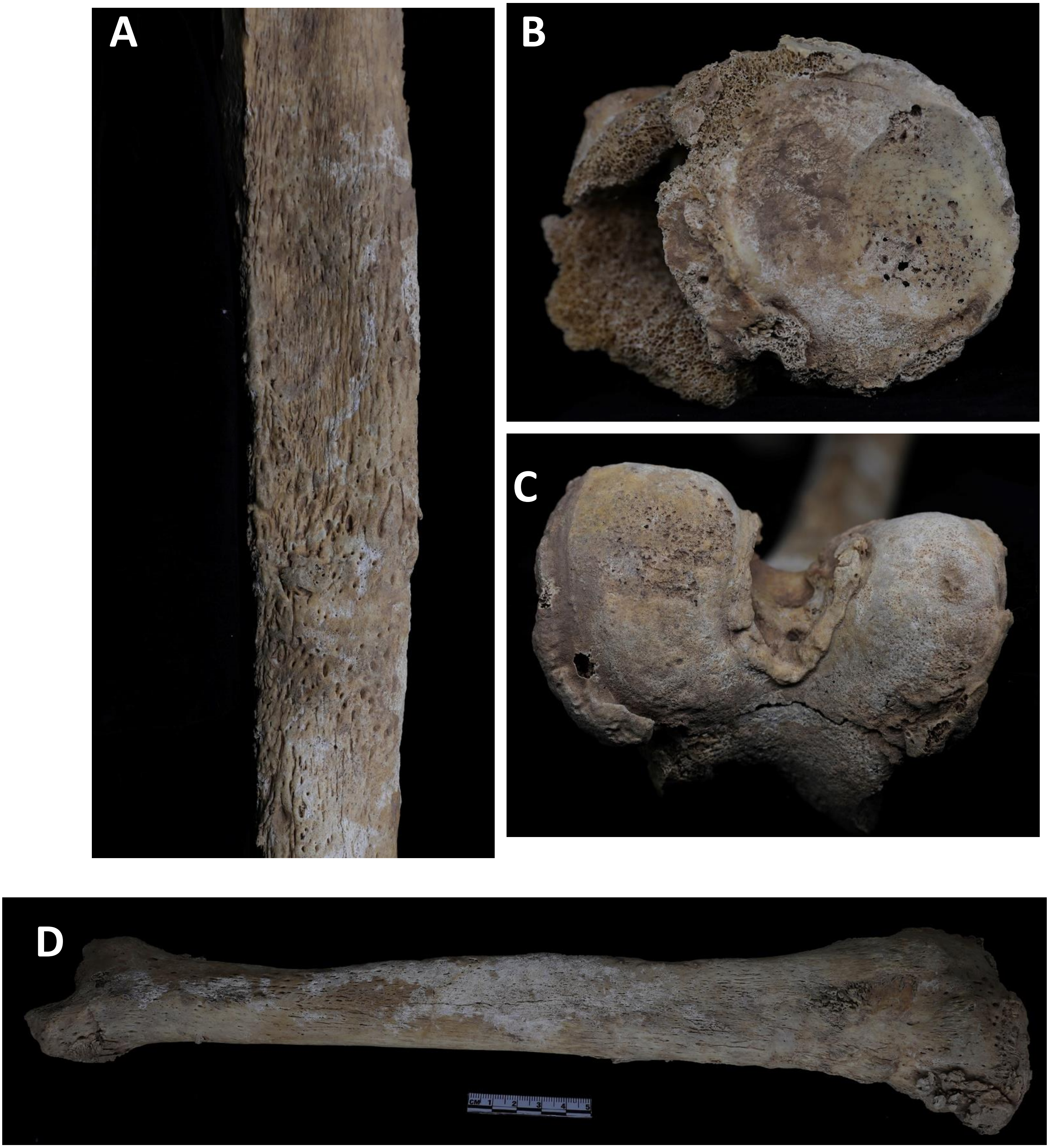

Supplement: Supplementary file 1 — Figure S1 Supplementary figures [file AJPA-178-530-s001.pdf]
