# Supplementary material for: Identifying treponemal disease in early East Asia
Source: Am J Biol Anthropol. 2022 Apr 27;178(3):530–43. doi: 10.1002/ajpa.24526 (PMC9545539; doi:10.1002/ajpa.24526)
Supplement: Supplementary file 2 — Table S1 Supplementary tables [file AJPA-178-530-s002.docx]

**Identifying treponemal disease in early East Asia.**

Supplementary Tables

*Supplementary Table 1. Inventory of skeletal elements excavated from Xingfulindai.*

| Skeletal element | Right | Left | Unknown | Total |
| --- | --- | --- | --- | --- |
| cranium |  |  |  | 236 |
| mandible |  |  |  | 199 |
| sternum |  |  |  | 52 |
| clavicle | 116 | 138 |  | 254 |
| scapula | 130 | 131 | 2 | 263 |
| humerus | 224 | 218 | 2 | 444 |
| radius | 121 | 130 | 1 | 252 |
| ulna | 166 | 171 |  | 337 |
| pelvis | 204 | 212 | 6 | 422 |
| femur | 327 | 351 | 3 | 681 |
| tibia | 305 | 315 | 2 | 622 |
| fibula | 157 | 166 | 7 | 330 |
| patella | 26 | 30 |  | 56 |
| calcaneus | 117 | 106 |  | 223 |
| talus | 134 | 126 |  | 260 |

*Supplementary Table 2. The number of present and affected skeletal elements from 12 Xingfulindai skeletons with lesions suggesting systemic pathology^*^.*

|  | sex | age (years) | cranium | humerus | radius | ulna | os coxa | femur | tibia | fibula | sets of foot bones |
| --- | --- | --- | --- | --- | --- | --- | --- | --- | --- | --- | --- |
| M26 | F | 18+ | present/hyperostosis | 2/0 | x | x | 2/0 | 2/0 | x | x | x |
| M84 | F | 20-24 | present/hyperostosis | 2/0 | 1/0 | 2/0 | 2/0 | x | 2/2 | x | 2/0 |
| M114 | U | 25-30 | present/hyperostosis | x | x | x | x | 2/0 | 2/2 | x | x |
| M173 | M | 35-50 | fragments/hyperostosis | 2/2 | 2/1 | 2/1 | 2/1 | 2/2 | 2/2 | 2/2 | 2/0 |
| M318 | U | 40+ | present/hyperostosis | x | x | x | x | x | x | x | x |
| M339 | M | 35-50 | x | x | x | x | 2/0 | 2/1 | 2/2 | x | x |
| M508 | M | 35+ | present/hyperostosis | x | x | x | x | x | 2/1 | x | x |
| M682 | F | 18+ | present/hyperostosis | x | x | x | x | x | x | x | x |
| M695 | F | 35-50 | present/caries sicca | 2/2 | 2/0 | 2/0 | 2/1 | 2/2 | 2/2 | 2/0 | 2/0 |
| M785 | U | 18+ | present/hyperostosis | 2/0 | x | x | 2/0 | 2/0 | 2/2 | x | x |
| M810 | U | 18+ | present/hyperostosis | 2/0 | x | x | 2/0 | 2/0 | x | x | x |
| M888 | U | 0.5-2 | present/SES | x | x | x | x | x | x | x | x |

* x – element is missing; # present/# affected, SES= Serpens endocrania symmetrica

*Supplementary Table 3. Diagnostic criteria for treponematosis modelled after Baker et al. (2020: Table 1) and presence of these changes in M173, M339, and M695 (P = pathognomonic, S = strongly suggestive, and C = consistent with).*

| **Lesion** | **Diagnostic strength** | **Clinical reference** | **Presence** |
| --- | --- | --- | --- |
| **Skull** |  |  |  |
| *Caries sicca* stages 1-3: clustered pits, confluent clustered pits and focal superficial cavitation | S | Hackett, 1976: 31-63 | M695 (frontal bone) |
| *Caries sicca* stages 4-5: circumvallate cavitation and radial scar | S | Hackett, 1976: 31-63 |  |
| *Caries sicca* stages 6-8: serpiginous cavitation, nodular cavitation, and caries sicca | P | Hackett, 1976: 31-63 | M695 (posterior calvarium) |
| Perforation of the palate and/or nasal bones | C/P | Hackett, 1976: 63-66 | M695 |
| Nasal cavity with smooth ‘bore-out’ tunnel passage | C/P | Hackett, 1976: 63-66 | - |
| Naso-palatine destruction with bone remodeling (gangosa) | P | Hackett, 1976: 63-66 | - |
| High palatal arch (congenital) | C | Harper et al., 2011: Table 2 | - |
| Saddle nose (congenital) | C | Ortner, 2003: 283 | - |
| **Teeth** |  |  |  |
| Hutchinson’s incisors (congenital syphilis) | P | Hackett, 1976: 441 | - |
| Moon’s molars (congenital syphilis) | P | Hackett, 1976: 441 | - |
| Fournier’s/  Mulberry molar | C | Ortner, 2003: 595 | - |
| **Long bones** |  |  |  |
| Periosteal reaction with plaques of new bone and/or striae | C | Hackett, 1976: 76-77 | M695  M173  M339 |
| Enlarged diaphysis and thickened cortex | C | Hackett, 1976: 79-97 | M695  M173 M339 |
| Saber-shin: pseudo-bowing of the tibia with antero-medial deposits of new bone with no bowing of the medullary cavity | S | Hackett, 1976: 100-101 | M339 |
| True tibial bowing (congenital syphilis) | C | Hackett, 1976: 101 | - |
| Nodes/Expansions rugose | S | Hackett, 1976: 79-97 | M695  M173 |
| Nodes/Expansions finely striated | S | Hackett, 1976: 79-97 | M695 |
| Nodes/expansion with focal cavitation (gummatous lesions) | P if bilateral; C if unilateral | Hackett, 1976: 79-97 | M695  M173 |
| Osteomyelitis (cloaca, sequestrum, involucrum, and enlarged bone) secondary to treponemal infection | C | Hackett, 1976: 91-93 | - |
| **Other bones** |  |  |  |
| Higoumenakis’ sign: unilateral enlargement of the sternal end of the clavicle (congenital) | C | Harper et al., 2011: Table 2 | - |
| Dactylitis (diaphyseal enlargement of foot or hand bones) | S | Ortner, 2003: 275 | - |
| Charcot joint: erosion, eburnation and destruction of weight bearing joints | C | Hackett, 1976: 106 | M339 |
| Sternal and vertebral erosion from aortic aneurysm pressure | C | Hackett, 1976: 106 | - |

*Supplementary Table 4. Diagnostic criteria for the differential diagnosis of the skeletal lesions discussed in the paper.*

|  | Pathogenesis in dry bone | Strongly suggestive | Consistent |
| --- | --- | --- | --- |
| Brucellosis *Brucella sp*. | Destructive lesions of the vertebral bodies and major joints (al-Shahed et al., 1994; Ortner, 2013; Roberts and Buikstra, 2019). | Circumscribed lytic lesions on anterior vertebral surface of lower thoracic and lumbar or sacroiliac joint (Ortner, 2013; Roberts and Buikstra, 2019). Sclerotic repair of lytic focus with anterior osteophytes (parrot’s beak) formation (Ortner, 2013; Roberts and Buikstra, 2019). | Lytic lesions on the surface of other major joints (ankle, knee and elbow) (al-Shahed et al., 1994; Ortner, 2013; Roberts and Buikstra, 2019); vertebral osteomyelitis (al-Shahed et al., 1994); vertebral ankylosis (al-Shahed et al., 1994; Ortner, 2013; Roberts and Buikstra, 2019). |
| Osteomyelitis Bone infections caused by pus-producing bacteria, including *Staphylococcus sp. and* *Streptococcus sp.* | Purulent infection of bone marrow with pus drained to the periosteum, causing vascular pressure and necrosis. Reactive bone, involucrum forms enveloping the necrotic bone. Pus drains through the involucrum often causing cloaca. Often localized (al-Shahed et al., 1994; Ortner, 2003; Roberts, 2019; Roberts and Buikstra, 2019). | Presence of involucrum, sequestrum, and cloacae; endosteal involvement (al-Shahed et al., 1994; Ortner, 2003; Roberts, 2019; Roberts and Buikstra, 2019). | Bone ankylosis of large joints. Severe formation of periosteal and endosteal bone (al-Shahed et al., 1994; Ortner, 2003; Roberts, 2019; Roberts and Buikstra, 2019). |
| Garre's sclerosing osteomyelitis  Bacterial infection is suspected | A form of chronic osteomyelitis caused by a mild infection, often linked to dental and ear infection. Non-suppurative inflammatory process with proliferative periostitis. Usually affects children and there is a predilection for males. The jaw is the most affected bone, followed by the tibia (Belli et al., 2002; Ortner, 2003; de Moraes et al., 2014; Liu et al., 2019). | Inflammation of the jaw with a characteristic “onion skin” (new bone depositions in a concentric way) radiographic appearance (Belli et al., 2002; Liu et al., 2019). | Periosteal reaction with deposits of new bone, cortical enlargement and obliteration of medullary canal. Periosteal formation of bone spicules. Lack of sequestrum and cloaca. (Belli et al., 2002; Ortner, 2003; Suma et al., 2007; de Moraes et al., 2014). |
| Treponematosis *Treponema pallidum* subspecies | *Treponema pallidum* subspecies  Generalised periostosis and osteitis can develop during earlier stages of this disease, but diagnostic lesions occur during the tertiary stage and tend to localize on bones with minimal tissue overlaying. Bone lesions tend to be symmetrical and affect multiple groups of bones (Baker et al., 2020; Hackett, 1975, 1976; Ortner, 2003; Harper et al., 2011). | *Treponema pallidum* subspecies  Generalised periostosis and osteitis can develop during earlier stages of this disease, but diagnostic lesions occur during the tertiary stage and tend to localize on bones with minimal tissue overlaying. Bone lesions tend to be symmetrical and affect multiple groups of bones (Baker et al., 2020; Hackett, 1975, 1976; Ortner, 2003; Harper et al., 2011).  . | Pseudo-bowing (saber shin) due to deposits of new bone on anterior part of the tibiae; true bowing (in the case of yaws); Charcot joint; non-gummatous widespread osteitis, with exostoses and periosteal and cortical thickening; symmetrical dactylitis (yaws and CS) (Baker et al. 2020; Hackett, 1975; 1976; Ortner, 2003; Harper et al. 2011). |
| Tuberculosis Mycobacterium tuberculosis and other bacteria of MTC complex | Focal lytic lesions with little or no bone regeneration and remodeling. Spine is the most affected skeletal area, often involving kyphosis of the bodies affected. The hip and knee are also often involved. Long bone lesions often lead to sequestra. Round lytic lesion on the skull that are more extensive in the inner table (Mosher et al., 2013; Ortner, 2003; Roberts and Buikstra, 2019). | Gibbus deformity.  Lytic destructions in the lower thoracic area involving 1 to 4 vertebral bodies with absence of bone formation (Ortner, 2003; Roberts and Buikstra, 2019). | A constellation of destructive focal lesions lacking reactive bone on ribs and surfaces of main joints (hip, knee and elbow) and periosteal reaction on long bones (Mosher et al., 2013; Ortner, 2003; Roberts and Buikstra, 2019). |
| Mycosis  Bone lesions can be caused by pathogenic fungi including genera *Cryptococcosis, Mucormycosis,* and *Aspergillosis* | *Aspergillosis* and *Mucormycosis* can cause destructive lesions of facial skeleton. Vertebral aspergillosis can cause destruction of vertebral bodies and gibbus deformity. *Cryptococcosis* causes lytic circumscribed lesions on the axial skeleton (Grauer and Roberts, 2019; Ortner, 2003). |  |  |
| Metabolic |  |  |  |
| Fluorosis  Caused by elevated concentrations of fluorine in drinking water | Enamel defects and discoloration. Mineralization of tendons and ligaments and bony excrescences at the point of muscular insertions. Generalized periosteal hyperostosis may occur (Brickley and Ives, 2008; Brickley and Mays, 2019; Littleton, 1999; Nelson et al., 2019; Ortner, 2003). | Formation of white patches on the enamel that later discolor into a brownish stain (Brickley and Mays, 2019; Littleton, 1999; Nelson et al., 2019; Ortner, 2003). Hypoplastic pitting of the enamel. Generalized proliferation of muscle and tendinous enthesophytes. Generalized osteophyte formation.  Generalized osteosclerosis (Brickley and Mays, 2019; Littleton, 1999; Nelson et al., 2019; Ortner, 2003). | Mineralization of interosseous ligaments of forearm and lower leg (Brickley and Ives, 2008; Brickley and Mays, 2019; Littleton, 1999; Nelson et al., 2019; Ortner, 2003). Ossification of spinal ligaments and marked osteophyte formation on vertebrae. Ankylosis of costovertebral joints. Calcification of major joints (Brickley and Ives, 2008; Brickley and Mays, 2019; Littleton, 1999; Nelson et al., 2019; Ortner, 2003). |
| Rickets  vitamin D deficiency | Lack of endochondral mineralization causing weakening and softening of bones. Metaphysis and growing plates are first affected. Skeletal involvement can be significant, but often weight bearing bones of the extremities and the rib cage are most affected (Brickley and Ives, 2008; Brickley and Mays, 2019; Snoddy et al., 2018). | Flaring and swelling of distal epiphysis and flattening of growing plates. Abnormal porosity of metaphyseal areas. Angulation of growing plates. Abnormal anterolateral bowing of distal metaphysis of long bones, more commonly of the lower limb (Brickley and Ives, 2008; Brickley and Mays, 2019; Snoddy et al., 2018). | Abnormal porosity and thinning of the skull. Costocartilage enlargement coupled with swelling or flaring; bowing of ribs. Characteristic “rachitic rosary” and “pigeon breast” deformity (Brickley and Ives, 2008; Brickley and Mays, 2019; Snoddy et al., 2018). |
| Neoplastic  Metastatic bone cancer  Uncontrolled cellular proliferation and dissemination of these cancer cells from their original site to the bone. | Only osteolytic metastatic cancers can mimic lesions observed in patients of treponemal disease. Langerhans cell histiocytosis, metastatic neoplasms, metastatic cancer of the skull or metastatic carcinoma are all plausible differential diagnostic (Hackett, 1976; Ortner, 2003). | | |
| Miscellaneous |  | | |
| Paget’s disease  Chronic skeletal disorder characterized by osteoclast-mediated bone destruction followed by excessive bone remodeling. The disease is rare in individuals younger than 40 years old but is the second most reported pathology affecting the bone. | Pathological increase in the bone remodeling rate resulting in a characteristic mosaic organization of the area of the bone affected (Burrell et al., 2019; Gennari et al., 2019; Ortner, 2003; Valenzuluela and Pietchmann, 2007). Early lesions are lytic in nature followed by sclerotic ones, thickening of the cortex and general bone enlargement of the region affected (Burrell et al., 2019; Gennari et al., 2019; Ortner, 2003; Valenzuluela and Pietchmann, 2007). Any region of the skeleton may be affected but with preference for the axial skeleton (Burrell et al., 2019; Gennari et al., 2019; Ortner, 2003; Valenzuluela and Pietchmann, 2007). | Mosaic structure of the bone visible with X-ray: trabecular coarsening, sclerotic areas, thickened cortex, and bone enlargement. Enlargement and bowing of long bones, especially weight bearing ones (Burrell et al., 2019; Gennari et al., 2019; Ortner, 2003; Valenzuluela and Pietchmann, 2007). | Macroscopic observation of bone enlargement, increased density and cortical thickening. Cotton wool appearance on the cranium, picture frame vertebrae and lytic wedge (Burrell et al., 2019; Gennari et al., 2019; Ortner, 2003; Valenzuluela and Pietchmann, 2007). |
